# IL-6–mediated endothelial injury impairs antiviral humoral immunity after bone marrow transplantation

**DOI:** 10.1172/JCI174184

**Published:** 2024-04-01

**Authors:** Ping Zhang, Peter Fleming, Christopher E. Andoniou, Olivia G. Waltner, Shruti S. Bhise, Jose Paulo Martins, Benjamin A. McEnroe, Valentina Voigt, Sheridan Daly, Rachel D. Kuns, Adaeze P. Ekwe, Andrea S. Henden, Alda Saldan, Stuart Olver, Antiopi Varelias, Corey Smith, Christine R. Schmidt, Kathleen S. Ensbey, Samuel R.W. Legg, Tomoko Sekiguchi, Simone A. Minnie, Mark Gradwell, Irma Wagenaar, Andrew D. Clouston, Motoko Koyama, Scott N. Furlan, Glen A. Kennedy, E Sally Ward, Mariapia A. Degli-Esposti, Geoffrey R. Hill, Siok-Keen Tey

**Affiliations:** 1 Translational Science and Therapeutics Division, Fred Hutchinson Cancer Center, Seattle, Washington, USA.; 2 QIMR Berghofer Medical Research Institute, Herston, Queensland, Australia.; 3 Infection and Immunity Program and Department of Microbiology, Biomedicine Discovery Institute, Monash University, Clayton, Victoria, Australia.; 4 Centre for Experimental Immunology, Lions Eye Institute, Nedlands, Western Australia, Australia.; 5 University of Queensland, St Lucia, Queensland, Australia.; 6 Royal Brisbane and Women’s Hospital, Herston, Queensland, Australia.; 7 Cancer Sciences Unit, Centre for Cancer Immunology, University of Southampton, Southampton, United Kingdom.; 8 Envoi Pathology, Brisbane, Queensland, Australia.; 9 Department of Pediatrics and; 10 Division of Medical Oncology, University of Washington, Seattle, Washington, USA.

**Keywords:** Transplantation, Bone marrow transplantation, Cytokines, Immunoglobulins

## Abstract

Endothelial function and integrity are compromised after allogeneic bone marrow transplantation (BMT), but how this affects immune responses broadly remains unknown. Using a preclinical model of CMV reactivation after BMT, we found compromised antiviral humoral responses induced by IL-6 signaling. IL-6 signaling in T cells maintained Th1 cells, resulting in sustained IFN-γ secretion, which promoted endothelial cell (EC) injury, loss of the neonatal Fc receptor (FcRn) responsible for IgG recycling, and rapid IgG loss. T cell–specific deletion of IL-6R led to persistence of recipient-derived, CMV-specific IgG and inhibited CMV reactivation. Deletion of IFN-γ in donor T cells also eliminated EC injury and FcRn loss. In a phase III clinical trial, blockade of IL-6R with tocilizumab promoted CMV-specific IgG persistence and significantly attenuated early HCMV reactivation. In sum, IL-6 invoked IFN-γ–dependent EC injury and consequent IgG loss, leading to CMV reactivation. Hence, cytokine inhibition represents a logical strategy to prevent endothelial injury, thereby preserving humoral immunity after immunotherapy.

## Abstract 2

Endothelial injury hastens IgG loss and cytomegalovirus reactivation after stem cell transplantation.

## Graphical abstract

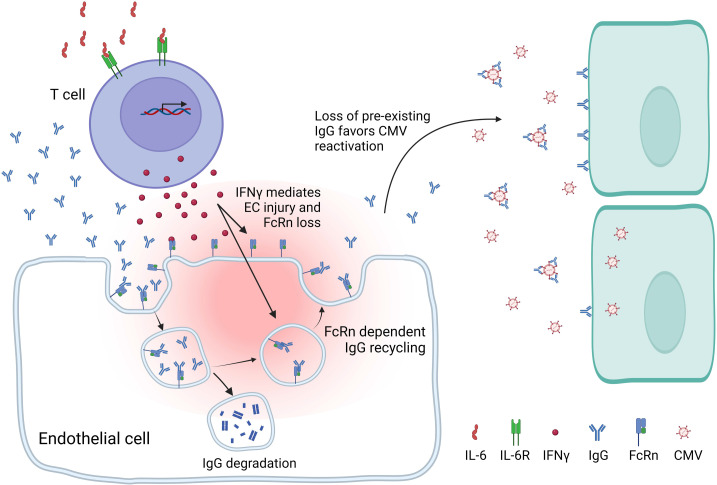

## Introduction

The influence of inflammation on various aspects of immune responses is recognized, yet the extent of these effects remains largely unknown. This is especially true within clinical contexts in which inflammation is invoked as part of the therapeutic process and represents a significant conceptual unknown, as well as an important clinical challenge. Allogeneic hematopoietic stem cell transplantation, also referred to as bone marrow transplantation (BMT), is an excellent therapeutic option for hematological malignancies and offers curative potential. The treatment is accompanied by significant inflammation resulting from the conditioning regimen and the development of graft-versus-host disease (GVHD).

Infectious complications, and in particular reactivation of the endemic herpesvirus CMV in the setting of BMT, can result in life-threatening disease and contribute significantly to early mortality (1–3). Cellular immunity has a well-established role in controlling CMV reactivation (4–6), but its recovery following BMT is significantly delayed by GVHD (7, 8), which induces a profound defect in antigen presentation by donor DCs and thus the priming of naive virus-specific T cells (5). Similarly, humoral immunity is critical for limiting CMV reactivation and spread, but BMT recipients experience prolonged defects in humoral immunity including low levels of Igs and reduced recovery of memory B cells, especially in patients who develop GVHD (9, 10). Historically, i.v. Ig therapy has been used extensively in these patients, but the half-life of IgG is considerably shortened after autologous and allogeneic BMT, and this is further exacerbated by GVHD (9, 11). How the inflammation induced during GVHD affects IgG longevity and the mechanisms involved remain unknown.

IgG homeostasis in vivo, including distribution and persistence, is regulated by the neonatal Fc receptor (FcRn). IgG constantly enter cells via fluid phase pinocytosis and is then recycled by the endosomal pathway to the cell surface bound to neonatal Fc receptor (FcRn); if not salvaged by FcRn, IgG is directed to lysosomes for degradation (12). While FcRn is expressed in a wide range of cells and tissues, endothelial cells (ECs) and hematopoietic cells (in particular, macrophages) have the highest levels of expression and represent the primary sites of IgG regulation (13), with the liver being the main site of IgG catabolism (14, 15).

Endothelial function and integrity are compromised after BMT, and this represents the pathogenic basis for a number of BMT-related complications, including sinusoidal obstructive syndrome and transplantation-associated thrombotic microangiopathy (TA-TMA). A recent study revealed an inverse association between the severity of TA-TMA and the half-life of administered antibodies (16), suggesting more significant impairment of IgG recycling in the presence of endothelial dysfunction.

Endothelial damage or dysfunction after BMT is attributable to multiple factors including GVHD, a multiphase pathogenic process in which tissue damage is mediated by pathogenic lymphocytes and their proinflammatory cytokines. IL-6 is a pleotropic cytokine that has a potent pathogenic role in various inflammatory conditions. It is the key dysregulated cytokine in BMT and plays a central role in GVHD pathogenesis (17, 18). We recently demonstrated that classical IL-6 signaling by donor T cells is the predominant pathway in GVHD pathogenesis (18), and inhibition of IL-6 signaling with the IL-6R monoclonal antibody tocilizumab has shown activity in the prevention of acute GVHD in clinical studies (19–21). Whether IL-6 contributes to endothelial damage and consequently affects IgG longevity is unknown.

Using the first mouse model of CMV reactivation after BMT, we recently showed that acute GVHD accelerates the loss of recipient CMV-specific IgG, resulting in pathogenic CMV reactivation (22). Here, we demonstrate that in BMT, IL-6–dependent inflammation caused EC injury with consequent impairment of FcRn-dependent IgG recycling resulting in loss of antiviral Igs and CMV reactivation. Interruption of IL-6R signaling preserved humoral immunity and attenuated CMV reactivation in both preclinical models and in patients after BMT. These studies reveal an additional, and to our knowledge, previously unknown mechanism of IgG loss after BMT relating to impaired IgG recycling. Furthermore, the findings provide definitive clinical evidence for the importance of CMV-specific antibodies in preventing CMV reactivation and reveal IL-6 inhibition as a logical strategy to preserve humoral immunity after immunotherapy.

## Results

### Murine CMV reactivation after BMT is IL-6 dependent.

**Figure 1:**
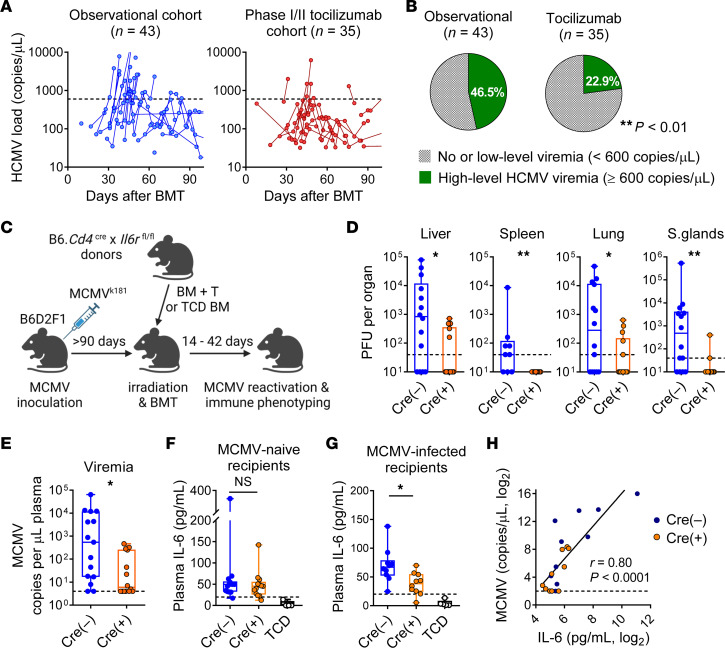
Inhibition of IL-6 signaling attenuates CMV reactivation in humans and mice. (**A**) HCMV viremia within 100 days of BMT in a prospectively enrolled observational cohort (left) and a phase I/II clinical trial investigating the addition of tocilizumab to standard GVHD prophylaxis (right). Only samples with detectable and quantifiable HCMV DNA are plotted, representing 25 of 43 and 20 of 35 at-risk patients, respectively. Dashed lines indicate the institutional threshold for preemptive antiviral therapy at 600 copies/μL. At-risk patients were those who were HCMV-seropositive and/or received a graft from an HCMV-seropositive donor. (**B**) Proportion of at-risk patients with clinically significant HCMV viremia in the 2 cohorts (**P < 0.01, by Fisher’s exact test). (**C**) Experimental schema of the murine BMT and MCMV reactivation model (created with BioRender.com). Latently MCMV-infected (**D**, **E**, **G**, and **H**) or uninfected (**F**) B6D2F1 mice were transplanted with BM (5 × 10^6^) and T cells (2 × 10^6^) from B6.*Cd4^Cre+^* *Il6r^fl/fl^* (Cre^+^) mice or littermate controls (Cre^–^). TCD BM (5 × 10^6^) from Cre^–^ donors was used in the non-GVHD control groups (TCD). (**D** and **E**) Viral loads in target organs and plasma (viremia) at weeks 4–5 after BMT (spleen: *n =* 9–10 per group from 2 experiments; others: *n =* 14–15 per group from 3 experiments). (**F**) Plasma IL-6 levels at week 3 in MCMV-naive recipients (GVHD groups: *n =* 13–14 per group from 3 experiments; TCD group: *n =* 4 from 1 experiment) and (**G**) Latently MCMV-infected recipients (GVHD groups: *n =* 9–10 per group from 2 experiments; TCD group: *n =* 5 from 1 experiment). (**H**) Correlation between plasma IL-6 levels and MCMV viremia 5 weeks after BMT (*n =* 9–10 per group from 2 experiments). Dashed lines indicate the limit of detection. Data are presented as the median ± IQR and were analyzed with the Mann-Whitney *U* test (*P < 0.05 and **P < 0.01).

We observed that patients enrolled in a phase I/II clinical trial evaluating the addition of tocilizumab to standard GVHD prophylaxis had lower rates of HCMV viremia requiring preemptive antiviral therapy (HCMV DNA ≥600 copies/μL) as compared with a prospectively enrolled observational cohort of patients who had undergone similar BMTs (19) (Figure 1, A and B). We therefore sought to determine the effects of IL-6 on CMV immunity and reactivation using our recently developed preclinical experimental systems (22).

The effects of clinical IL-6 inhibition can be modeled in preclinical murine transplant models using a CD4-Cre system to ablate the IL-6 receptor (IL-6R) and, consequently, classical IL-6–mediated signaling in both CD4^+^ and CD8^+^ donor T cells (Figure 1C) (18, 23). In these transplants, recipient mice were latently infected with murine CMV (MCMV). MCMV reactivation, as measured by viral loads in target organs (liver, spleen, lungs, and salivary glands) and plasma viremia, was reduced in the absence of IL-6R signaling in donor T cells (Figure 1, D and E). We next measured systemic IL-6 in the presence or absence of CMV reactivation. T cell–specific deletion of IL-6R had no effect on plasma levels of IL-6 in the absence of CMV (Figure 1F). However, IL-6 levels were lower in recipients of *Il6r^–/–^* T cells in the context of MCMV infection (Figure 1G), and there was a direct correlation between IL-6 levels and MCMV viremia (Figure 1H). Thus, T cell–specific IL-6R deletion did not directly affect IL-6 levels, whereas changes in systemic IL-6 levels reflected the magnitude of MCMV reactivation.

### T cell–specific ablation of IL-6R attenuates MCMV reactivation by preserving recipient-derived, MCMV-specific IgG.

**Figure 2:**
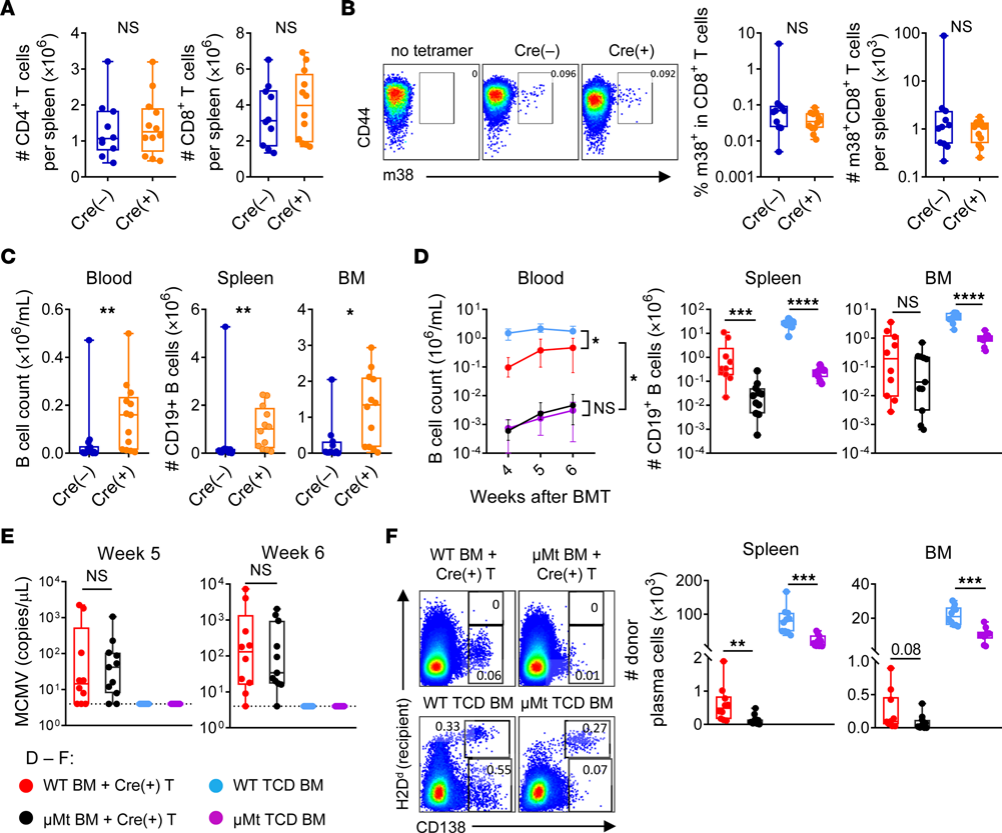
IL-6 signaling does not modify protective donor T cell or B cell responses to MCMV. (**A**–**C**) Latently infected B6D2F1 mice were transplanted with BM (5 × 10^6^) and T cells (2 × 10^6^) from B6.*Cd4^Cre+^* *Il6r^fl/fl^* (Cre^+^) mice or littermate controls (Cre^–^) and analyzed 4–5 weeks after BMT. (**A**) Numbers of CD4^+^ and CD8^+^ T cells in spleens (*n =* 10–12 from 3 experiments). (**B**) m38 tetramer^+^ CD8^+^ T cells in spleens (*n =* 11–12 from 3 experiments) including representative flow cytometric plots. (**C**) CD19^+^ B cells in the blood, spleen or BM (*n =* 11–14 from 3 experiments; BM was from femur and tibia). (**D**–**F**) Latently infected B6D2F1 mice were transplanted with BM (5 × 10^6^) from C57BL/6J (WT) mice or with B6.μMt BM and T cells (2 × 10^6^) from B6.*Cd4^Cre+^* *Il6r^fl/fl^* (Cre^+^) mice. TCD BM (5 × 10^6^) was administered to non-GVHD control groups (*n =* 10–11 per group from 2 experiments). (**D**) CD19^+^ B cells in the peripheral blood over time or in the spleen and BM (femur and tibia for BM) at week 6 after BMT. (**E**) MCMV viremia at weeks 5 and 6 after BMT. (**F**) Representative flow cytometric plots (CD90.2^–^CD11b^–^CD19^–^ gate) showing CD138^+^ plasma cells in the spleen and BM at week 6 without a discernible persistent recipient (H2D^d+^) population in the BM+T settings. Number of CD19^–^CD138^+^ plasma cells in the spleen and BM (femur and tibia) at week 6. Data are presented as the median ± IQR and were analyzed with the Mann-Whitney *U* test (*P < 0.05 and **P < 0.01).

Virus-specific T cells are important in controlling CMV reactivation after BMT (4, 5), therefore, we examined T cell responses generated in the absence of IL-6R signaling. We found that the reconstitution of splenic CD4^+^ and CD8^+^ T cells was comparable in the presence or absence of IL-6R signaling (Figure 2A). We observed that MCMV-specific CD8^+^ T cells, quantified by the m38 tetramer, were present at low levels prior to MCMV reactivation (Supplemental Figure 1A; supplemental material available online with this article; https://doi.org/10.1172/JCI174184DS1), and their levels were not different between the groups at later time points (Figure 2B). Likewise, MCMV-specific CD4^+^ T cell responses were not detectable prior to reactivation and only present at low frequencies, with no increases seen in the recipients of *Il6r^–/–^* T cells at the onset of viremia (Supplemental Figure 1, B and C). Hence, disruption of IL-6 signaling within T cells did not enhance the development of MCMV-specific T cells, which is known to be severely abrogated by GVHD (5, 8, 24).

Follicular helper T (Tfh) cells are required for germinal center B cell differentiation and are sustained by intrinsic IL-6 signaling (25). As expected, the expression of Tfh markers was lower in *Il6r^–/–^* T cells (Supplemental Figure 2, A and B). However, recipients of *Il6r^–/–^* T cells showed improved donor B cell recovery (Figure 2C). The transplantation of B6.μMt BM (Supplemental Figure 2C), incapable of generating mature B cells or plasma cells (26), resulted in significantly lower B cell numbers (Figure 2D) but did not affect MCMV reactivation (Figure 2E). Class-switched and germinal center B cells (Supplemental Figure 2, D and E), as well as donor-derived plasma cells (Figure 2F), showed limited recovery in the first 6 weeks after BMT in a GVHD setting. Collectively, these data demonstrate that donor-derived B cells and plasma cells did not contribute to MCMV control early after BMT.

**Figure 3:**
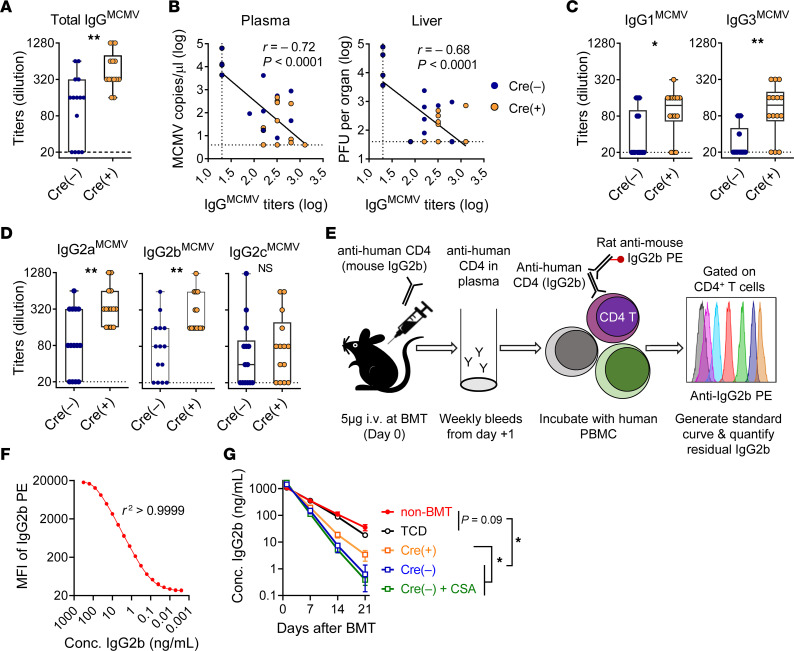
IL-6 signaling promotes the loss of recipient IgG. (**A**–**D**) Latently infected B6D2F1 recipient mice were transplanted with BM (5 × 10^6^) and T cells (2 × 10^6^) from uninfected B6.*Cd4^Cre+^* *Il6r^fl/fl^* (Cre^+^) mice or littermate controls (Cre^–^) and analyzed 4–5 weeks after BMT (*n =* 14 per group from 3 experiments). (**A**) MCMV-specific IgG titers. (**B**) Correlation between MCMV-specific IgG titers and viral load in plasma and liver. (**C** and **D**) Titers of MCMV-specific IgG1 and IgG3 (**C**) and IgG2 allotypes (**D**). Dashed lines indicate limit of detection. (**E**) Experimental schema for the quantification of IgG clearance by flow cytometry and (**F**) representative standard curve. (**G**) Noninfected B6D2F1 mice were transplanted with *Cd4^Cre+^* *Il6r^fl/fl^* BM (5 × 10^6^) plus T cells (2 × 10^6^) (Cre^+^, *n =* 10, orange) or with *Cd4^Cre–^* *Il6r^fl/fl^* (Cre^–^) BM plus T cells from littermate control mice, the latter with or without CSA to limit GVHD (Cre^–^, *n =* 12, blue; or Cre^–^ plus CSA, *n =* 8, green). Mice transplanted with TCD *Cd4^Cre–^* *Il6r^fl/fl^* BM alone (TCD, *n =* 5, black) and nontransplanted mice (non-BMT, *n =* 6, red) were used as controls. The half-life (from days 7–21) of administered IgG2b was calculated for each individual mouse and compared between the indicated groups. Conc., concentration. Data were combined from 2 experiments. Data re presented as the median ± IQR and were analyzed with the Mann-Whitney *U* test (*P < 0.05 and **P < 0.01).

MCMV-infected mice transplanted with MCMV-naive *Cd4^Cre+^* *Il6r^fl/fl^* donor grafts had significantly higher titers of MCMV-specific IgG compared with those transplanted with WT littermate donor grafts (Figure 3A). MCMV-specific IgG titers were significantly and negatively correlated with MCMV load in plasma and liver (a key target organ of MCMV) (Figure 3B), demonstrating the importance of recipient-derived humoral immunity in this context (22). Differences in MCMV-specific IgG levels were observed for IgG1, IgG2, and IgG3 (Figure 3, C and D). The IgG2 allotype was particularly informative, as IgG2a can only be produced by the recipient strain (B6D2F1) (27). Thus, recipient-derived IgG was preserved in hosts receiving *Il6r^–/–^* T cells. Next, we determined whether improved survival of recipient B cells or plasma cells could account for the persistence of recipient CMV-specific IgG observed in mice transplanted with a *Il6r^–/–^* T cell graft. In the presence of T cells, both B cells and plasma cells were rapidly eliminated after BMT, independent of IL-6R signaling (Supplemental Figure 2, F–I); thus, recipient B cells or plasma cells made no contribution to the recipient IgG pool differences associated with IL-6 signaling. As previously noted (22), loss of recipient plasma cells and B cells is significantly delayed in the absence of donor T cells and GVHD. Collectively, these data demonstrate that, although the absence of IL-6R signaling preserved recipient CMV-specific IgG, it did not affect the elimination of recipient IgG-producing cells.

Having established a correlation between IL-6 signaling in donor T cells and the preservation of recipient-derived, MCMV-specific IgG, we investigated the effect of IL-6 on the pharmacokinetics of IgG. The exogenous administration of monoclonal mouse IgG2b on day 0 of BMT followed by flow cytometric quantification allowed for the evaluation of IgG pharmacokinetics (Figure 3, E and F) (28). The loss of administered IgG2b was more rapid (*t_1/2_* 1.6–2.1 days) for the transplanted mice as compared with the nontransplanted (non-BMT) mice (*t_1/2_* 4.1 days), or for mice transplanted with T cell–depleted (TCD) BM (*t_1/2_* 3.8 days), which did not develop GVHD (Figure 3G and Supplemental Figure 3A). Among the transplanted mice, the half-life was shorter in recipients of *Cd4^Cre–^* *Il6r^fl/fl^* grafts as compared with recipients of *Il6r^–/–^* (Cre^+^) grafts (*t_1/2_* 1.6 days vs. 2.1 days), and the difference was maintained even when GVHD was attenuated by the administration of cyclosporine A (CSA) (*t_1/2_* 1.7 days) (Figure 3G and Supplemental Figure 3A), a standard GVHD prophylaxis agent in clinical BMT. Since classical IL-6 signaling promotes Th17/Tc17 differentiation (18), we examined the effect of T cell–derived IL-17 and found that IL-17A deficiency in donor T cells did not alter IgG pharmacokinetics (Supplemental Figure 3B). Together, these data demonstrate that inhibition of IL-6 signaling in T cells reduced antibody turnover and preserved recipient-derived IgG, thereby attenuating MCMV reactivation.

### Rapid loss of IgG after BMT is due to endothelial injury that is attenuated in the absence of IL-6R signaling in T cells.

**Figure 4:**
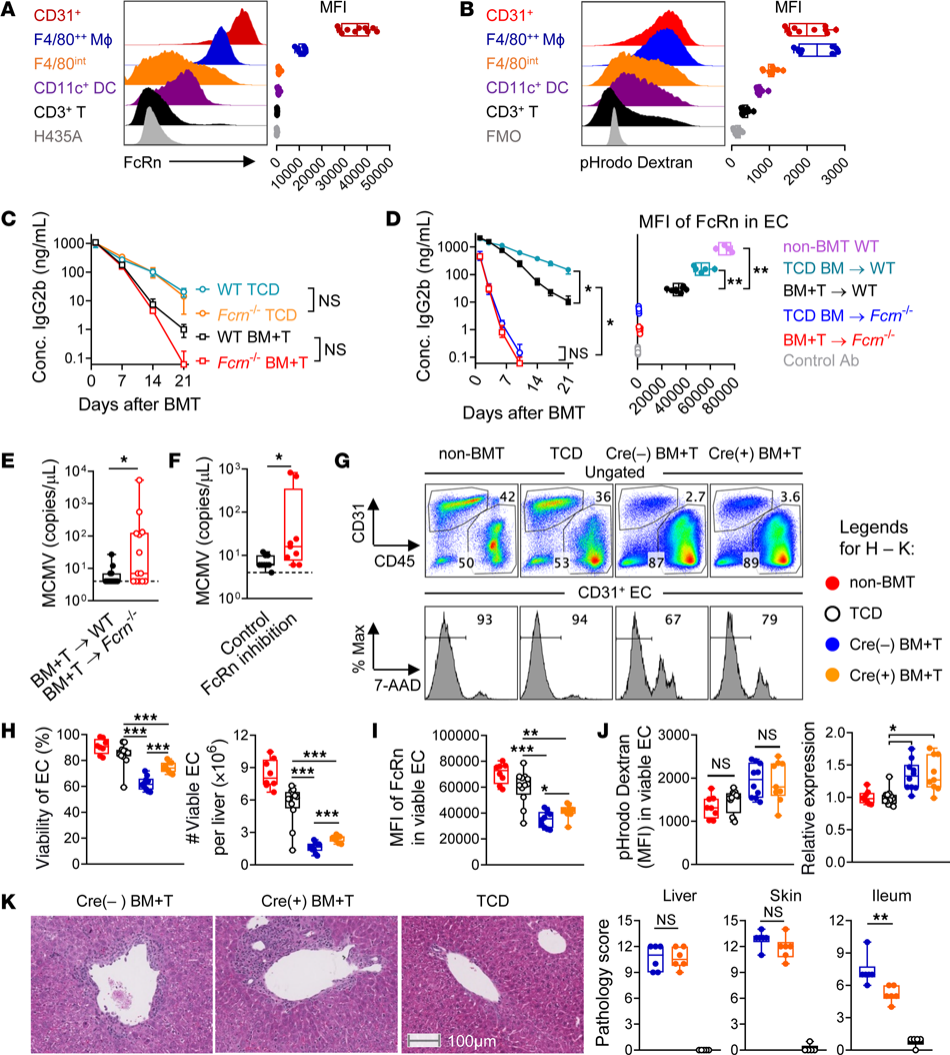
EC injury impairs FcRn recycling and promotes IgG loss. (**A** and **B**) Noninfected B6D2F1 were transplanted with BM (5 × 10^6^) and T cells (2 × 10^6^) from *Cd4^Cre–^* *Il6r^fl/fl^* donors (*n =* 10 from 2 experiments). On day 14, liver cells were analyzed for (**A**) the expression of FcRn (i.e., representing IgG recycling) and (**B**) endocytosis (i.e., representing IgG uptake); representative histograms are shown. (**C**) Noninfected B6D2F1 mice were transplanted with BM (5 × 10^6^) with or without T cells (2 × 10^6^), and the half-life of infused IgG2b was compared between the indicated groups (*n =* 5, 5, 8, and 8). (**D**) Uninfected WT or *Fcrn^–/–^* mice were transplanted with BM (10 × 10^6^) with or without T cells (5 × 10^6^) from BALB/c donors. The kinetics of administered IgG2b is shown (left), and the half-life was compared between the indicated groups (*n =* 4, 4, 8, and 7). Liver ECs were analyzed for the expression (MFI) of FcRn (right) on day 21. (**E**) MCMV latently infected *Fcrn^–/–^* or WT B6 mice were transplanted with BM (10 × 10^6^) plus T cells (3 × 10^6^) from BALB/c donors, and MCMV viremia was quantified on day 19 (*n =* 13–14 per group from 2 experiments). (**F**) Latently MCMV-infected B6D2F1 mice were transplanted with BM (5 × 10^6^) plus T cells (2 × 10^6^) from B6 donors and treated with a FcRn inhibitor or a control antibody. MCMV viremia was quantified on day 21 (*n =* 9 per group from 2 experiments). (**G**–**J**) Noninfected B6D2F1 mice were transplanted with BM (5 × 10^6^) with or without T cells (2 × 10^6^) from *Cd4^Cre^* *Il6r^fl/fl^* donors, and livers were analyzed on day 14 (*n =* 8–12 from 2 experiments). (**G**) Representative flow cytometric plots showing frequencies and viability of CD31^+^ ECs. Max, maximum. (**H**) Viability and number of ECs per liver. (**I**) FcRn expression (MFI). (**J**) MFI of pHrodo dextran (left) with normalized expression relative to the non-BMT group (right). (**K**) Noninfected B6D2F1 mice were transplanted with BM (5 × 10^6^) with or without T cells (2 × 10^6^) from *Cd4^Cre^* *Il6r^fl/fl^* (Cre^+^) or littermate control (Cre^–^) donors. GVHD target organs were taken for histological analysis on day 14. Shown are representative images of liver sections (left) and pathology scores in liver, skin, and ileum (right). Scale bar: 100 μm. Data are presented as the median ± IQR and were analyzed with the Mann-Whitney *U* test (*P < 0.05, **P < 0.01, and ***P < 0.001).

IgG homeostasis is regulated by FcRn, which is highly expressed on ECs and macrophages. Since the liver represents a primary site of IgG regulation, we isolated CD31^+^ ECs and hematopoietic cells from liver (29) after BMT and evaluated the pathways that affect IgG metabolism, namely, the expression of FcRn and endosomal-lysosomal activity. ECs and macrophages had the highest level of functional FcRn after BMT, as measured by their uptake of a mutated human IgG (MST-HN), which binds FcRn with high affinity (Figure 4A) (13, 30). We next conducted an endocytosis assay, which represents a combination of endocytic/pinocytic uptake and degradative activity of endosomes, and found this to be most prominent in ECs and macrophages (Figure 4B). Thus, ECs and macrophages represented significant sites of IgG regulation after BMT. Next, we analyzed the contribution of FcRn-dependent IgG recycling to IgG pharmacokinetics in the context of BMT by transplanting *Fcrn^–/–^* or WT grafts. Transplantation of *Fcrn^–/–^* grafts did not have a significant effect on the pharmacokinetics of IgG (Figure 4C). In contrast, FcRn deficiency in recipient cells significantly reduced the half-life of IgG (Figure 4D, left panel). This was associated with an absence of FcRn expression in ECs (Figure 4D, right panel). Of note, hematopoietic cells, including macrophages in the liver, had low levels of recipient chimerism from day 14 in all bone marrow plus T cell (BM+T) transplant models (Supplemental Figure 4, A–C), thus excluding any confounding effects from recipient macrophages. *Fcrn^–/–^* recipients demonstrated significantly higher MCMV reactivation early after BMT (Figure 4E). Similar results were obtained when endogenous FcRn activity was blocked with an antagonist that was biosimilar to efgartigimod (30). Temporary FcRn inhibition in recipients in which preexisting CMV-specific IgG was normal exacerbated MCMV reactivation after BMT (Figure 4F). Collectively, these data demonstrate that ECs were the primary regulator of IgG recycling after BMT in a process that was critically dependent on FcRn. Notably lack of FcRn activity led to CMV reactivation.

Next, we examined whether IL-6 signaling affects FcRn expression or endocytosis in liver ECs. Consistent with the pharmacokinetics of IgG, the transplantation of T cell–replete (BM+T) grafts was associated with significantly lower liver EC viability, numbers, and FcRn expression, all of which were significantly attenuated by IL-6R ablation in donor T cells (Figure 4, G–I). The endosomal-lysosomal pathway in ECs was also more active in BM+T groups, but this was not altered by IL-6R expression on donor T cells (Figure 4J). Hence, rapid IgG loss after BMT in the presence of T cells was attributable to a combination of impaired FcRn-dependent IgG recycling and increased IgG uptake and degradation in ECs, but the protective effect of IL-6R ablation in T cells was dependent solely on the FcRn pathway. IL-6R ablation did not affect the infiltration (Supplemental Figure 4, D and E), FcRn expression (Supplemental Figure 4F), or endocytic activity (Supplemental Figure 4G) of macrophages, further supporting the central role of ECs in regulating IgG kinetics after BMT. Importantly, T cell–specific IL-6R ablation resulted in a consistent improvement in EC viability and FcRn activity that was maintained over time (Supplemental Figure 4H). In order to understand whether the preservation of EC and FcRn integrity in recipients of *Il6r^–/–^* T cells was due to reduced GVHD, we analyzed GVHD target organ pathology, focusing on the liver, where most EC injury is observed. We performed this analysis on day 14 after BMT, when a reduction in the clearance of recipient IgG was already established in recipients of *Il6*r*^–/–^* T cells. IL-6 signaling did not affect GVHD severity in either the liver or skin at this time point (Figure 4K), while there was a modest effect on pathology in the small intestine. This is consistent with our previous findings in which the effects of IL-6 signaling on GVHD occurred beyond day 14 in this system (18), at a time when the changes in EC integrity and IgG clearance have already occurred. Thus, the protection from EC injury and recipient IgG loss in the absence of IL-6 signaling after BMT was not due to the inhibition of GVHD per se, but rather was a secondary consequence of IL-6 signaling in donor T cells. Collectively, these data demonstrate that T cell–specific ablation of IL-6R extended the half-life of recipient-derived IgG after BMT by reducing EC injury and maintaining FcRn-dependent IgG recycling.

### IL-6 promotes EC injury by maintaining IFN-γ expression in donor T cells.

**Figure 5:**
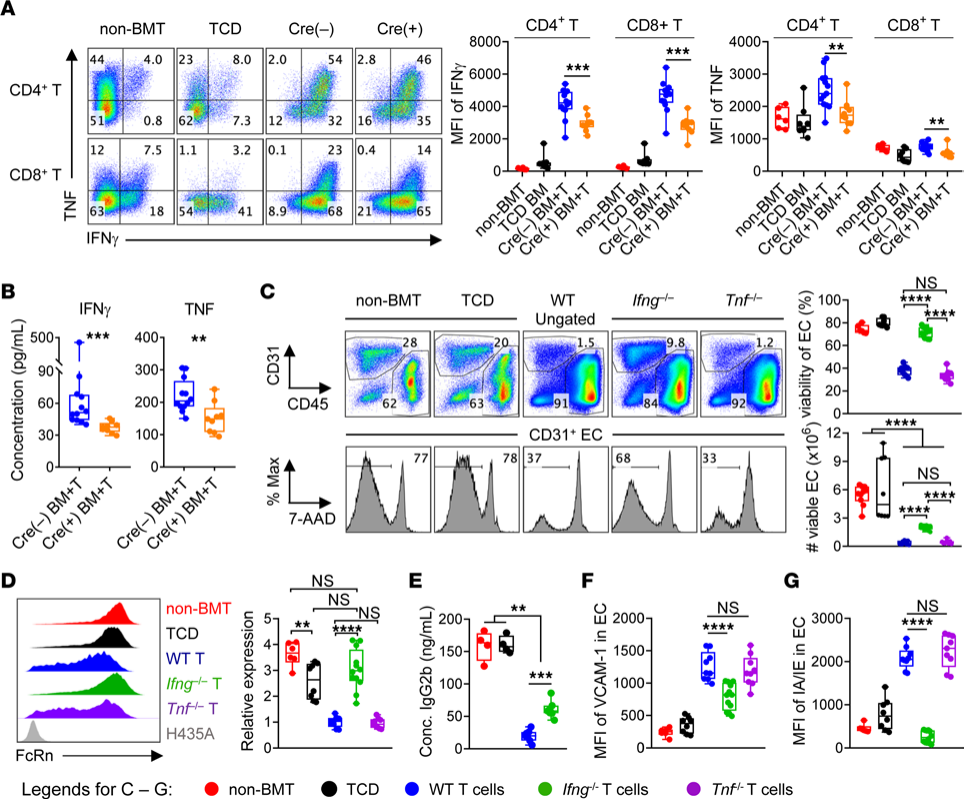
IL-6 maintains Th1 responses and IFN-γ secretion which mediates EC injury. (**A** and **B**) Noninfected B6D2F1 mice were transplanted with BM (5 × 10^6^), with or without T cells (2 × 10^6^), from *Cd4^Cre^* *Il6r^fl/fl^* (Cre^+^) or littermate control (Cre^–^) donors. (**A**) Expression of IFN-γ and TNF in splenic T cells on day 14 (*n =* 6, 8, 10, and 13 from 2 experiments), as shown by representative flow plots and MFI. (**B**) Plasma levels of IFN-γ and TNF on day 14 (*n =* 12, 9 per group from 2 experiments). (**C** and **D**) Noninfected B6D2F1 were transplanted with TCD BM (5 × 10^6^) from C57BL6J (WT) ± T cells (2 × 10^6^) from WT, *Ifng^–/–^*, or *Tnf^–/–^* donors (*n =* 6–12 from 2 experiments). On day 12, liver ECs were analyzed for (**C**) viability and numbers per liver and (**D**) expression of FcRn (MFI) relative to WT T group. (**E**) Noninfected B6D2F1 were transplanted with TCD BM (5 × 10^6^) from C57BL6J (WT) mice with or without T cells (2 × 10^6^) from WT or *Ifng^–/–^* donors, together with mouse anti–human CD4 IgG2b. Plasma levels of infused Abs were determined on day 12 (*n =* 4, 4, 7, 7 per group); non-BMT controls were included for comparison. (**F** and **G**) Experiments were conducted as described in **C** and **D** and analyzed for the expression of (**F**) VCAM-1 and (**G**) MHC-II on liver ECs. Data are presented as the median ± IQR and were analyzed with the Mann-Whitney *U* test (**P < 0.01, ***P < 0.001, and ****P < 0.0001).

Having excluded the contributions of Tfh and the IL-17 pathway in the protective effect of IL-6 inhibition, we examined its effect on Th1/Tc1 differentiation and potential downstream effects on EC injury and FcRn expression. T cell–specific IL-6R ablation was associated with significantly lower expression of IFN-γ and TNF in T cells in the spleen and liver (Figure 5A and Supplemental Figure 5, A and B) and reduced cytokine levels in the blood (Figure 5B). Consistent with reduced cytokine production, *Il6r^–/–^* T cells demonstrated lower levels of T-bet expression in both spleen and liver (Supplemental Figure 5, C and D). Of note, IL-6R ablation did not affect the infiltration of donor T cells into the liver (Supplemental Figure 5E). In contrast to IL-6R ablation, CSA had no effect on systemic levels of IFN-γ or TNF (Supplemental Figure 5F).

To investigate the contribution of IFN-γ and TNF in EC injury and function after BMT, we transplanted WT TCD BM together with WT, *Ifng^–/–^* or *Tnf^–/–^* T cells into allogeneic recipients. T cell–specific deletion of IFN-γ resulted in reduced EC injury (Figure 5C) and maintenance of FcRn expression in ECs, comparable to that seen in nontransplanted recipients (Figure 5D). As expected, the maintenance of IgG levels was also increased in the absence of IFN-γ (Figure 5E). Furthermore, T cell–specific deletion of IFN-γ also correlated with reduced expression of vascular cell adhesion protein 1 (VCAM-1) (Figure 5F), a marker of endothelial activation and injury (31), and of MHC class II (Figure 5G), a marker of endothelial activation. In contrast, deficiency of TNF in T cells or blockade of TNF signaling in recipient tissues did not protect against EC injury or FcRn loss (Figure 5, C, D, F, and G, and Supplemental Figure 5, G and H). We further examined cytokine expression in T cells in the liver at a later time point (day 21). *Il6r^–/–^* T cells showed significantly reduced IFN-γ secretion, with a modest reduction in TNF only observed in CD4^+^ T cells (Supplemental Figure 5, I and J). Interestingly, IFN-γ expression was inversely associated with EC injury and FcRn expression (Supplemental Figure 5, K and L). Thus, T cell–derived IFN-γ mediates EC injury and loss of FcRn, which is attenuated in the absence of IL-6 signaling. These results reveal a mechanism by which IL-6R inhibition, but not calcineurin inhibition with CSA, protected against IgG loss.

### Endothelial injury during GVHD is characterized by an IFN-γ– and JAK/STAT-dependent inflammatory signature.

**Figure 6:**
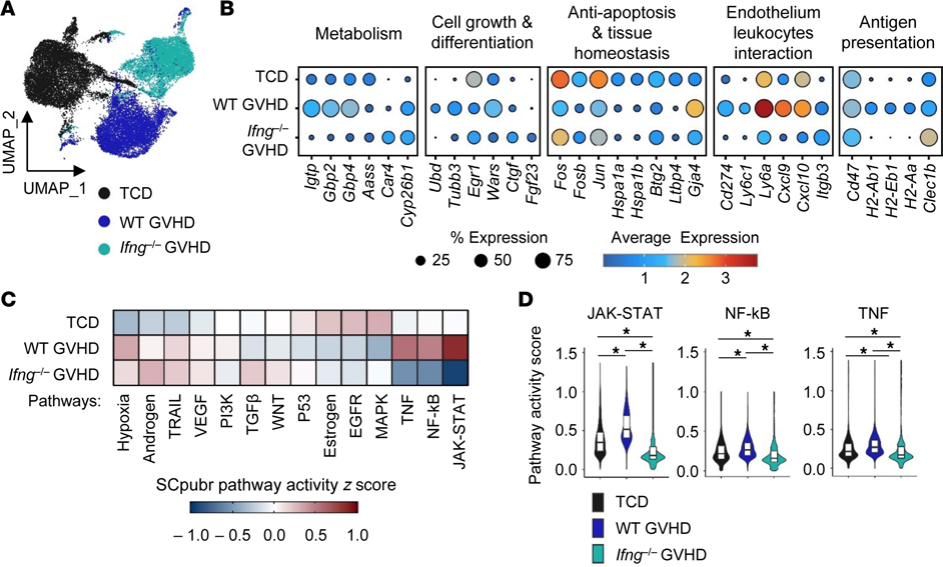
Endothelial injury during GVHD is characterized by an IFN-γ and JAK/STAT-dependent inflammatory signature. Noninfected B6D2F1 recipient mice were transplanted with WT TCD BM (5 × 10^6^) with or without T cells (2 × 10^6^) from WT or *Ifng^–/–^* donors. ECs from 3 mice per group were isolated from the liver on day 7 and processed for single-cell RNA-Seq. (**A**) Uniform manifold approximation and projection (UMAP) of ECs colored by groups. (**B**) Expression of genes across groups. (**C**) SCpubr pathway activity scores (*z* score scaled) across groups. (**D**) Violin plots of pathway activity scores (JAK/STAT, NF-κB, and TNF), with each group’s mean compared using a Wilcoxon test (*P < 0.001).

To examine the effect of IFN-γ on ECs, we performed single-cell RNA-Seq on liver ECs at day 7 after BMT, a time when IgG loss begins, but before extensive endothelial loss occurs. We performed these analyses in GVHD groups receiving WT or *Ifng^–/–^* T cells and a non-GVHD group receiving TCD grafts. Dimension reduction approaches revealed distinct clusters in each of the 3 groups (Figures 6A and Supplemental Figure 6, A and B). The predominant differentially expressed genes in GVHD versus non-GVHD groups were those involved in EC-leukocyte interactions, MHC class II expression, allograft rejection, and type I and II IFN responses (Figure 6, B and C, and Supplemental Figure 6C). Expression of these genes was markedly suppressed in the GVHD recipients receiving *Ifng^–/–^* T cells, as were those involved in JAK/STAT, NF-κB, and TNF signaling pathways (Figure 6, B and C, and Supplemental Figure 6C). Of note, the SCpubr JAK/STAT (Figure 6D), and HALLMARK IFN-γ pathways (Supplemental Figure 6D) were significantly augmented by GVHD but absent in GVHD recipients receiving *Ifng^–/–^* T cells. These data provide an atlas of endothelial injury after allogeneic BMT and demonstrate the pivotal role for IFN-γ in promoting local T cell interactions and associated damage.

### IL-6R inhibition with tocilizumab reduces HCMV viremia following allogeneic BMT in association with enhanced HCMV-specific humoral immunity.

**Figure 7:**
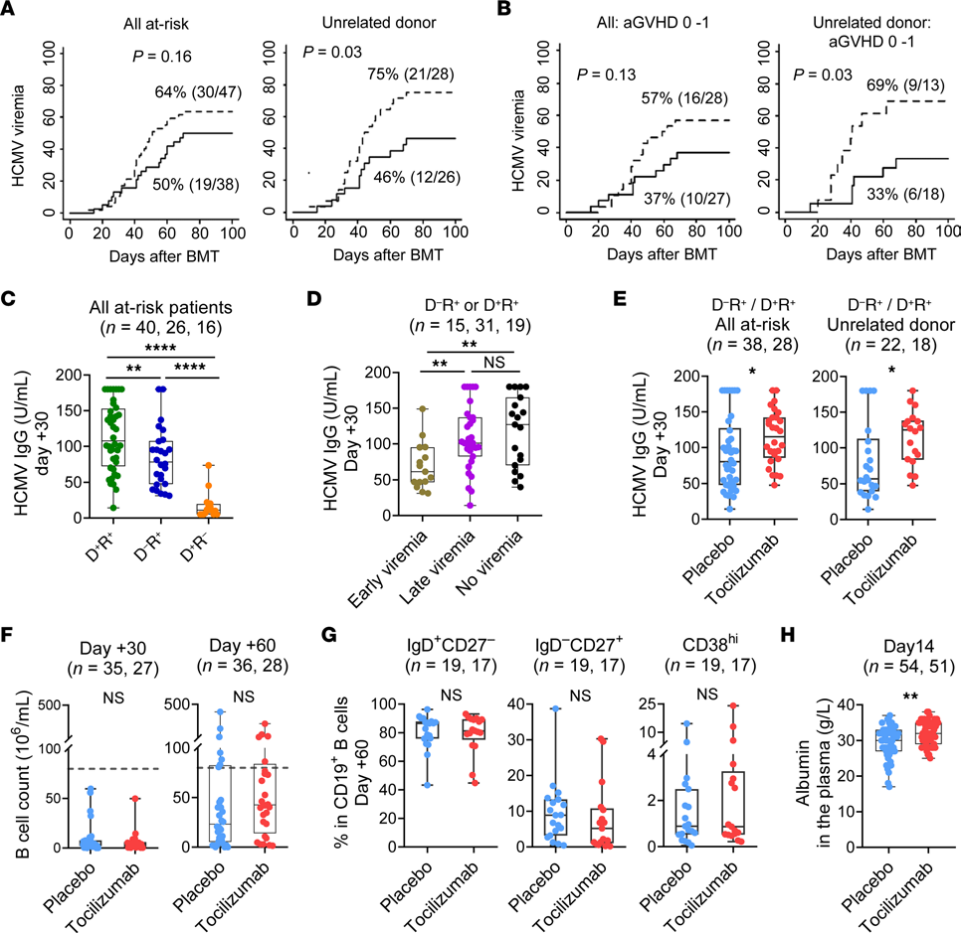
IL-6R inhibition with tocilizumab maintains CMV-specific humoral immunity and protection from early HCMV reactivation. Participants of a phase III clinical trial who were enrolled at Royal Brisbane and Women’s Hospital and at risk for HCMV reactivation were included for analysis (*n =* 85). (**A** and **B**) Cumulative incidence of HCMV viremia in all at-risk patients (*n =* 85) and a subset of patients who received unrelated donor graft (*n =* 54). Broken lines represent the placebo arm, and solid lines represent the tocilizumab arm. (**B**) Cumulative incidence of HCMV viremia among patients who had grade 0–1 acute GVHD (*n =* 55) and a subset of patient who received an unrelated donor graft (*n =* 31). (**C**–**E**) Plasma HCMV-specific IgG levels were determined at day 30 after BMT in at-risk patients (*n =* 82; 1 patient was excluded because of HCMV viremia prior to day 0, and 2 patients did not have a plasma sample stored). (**C**) Correlation between day 30 HCMV IgG levels and donor (D) and recipient (R) serostatus. (**D**) Correlation between day 30 HCMV IgG levels and HCMV viremia among HCMV-seropositive recipients. Patients were categorized according to early viremia (within the first 5 weeks after BMT), late viremia (from weeks 5–14 after BMT), and no viremia (no detectable viremia within the first 14 weeks, after which HCMV monitoring was no longer routinely performed. (**E**) Correlation between day 30 HCMV IgG levels and the treatment arm among HCMV-seropositive recipients and the subset receiving an unrelated donor graft (right). (**F**) CD19^+^ B cell counts in the peripheral blood 30 and 60 days after BMT in HCMV-seropositive recipients. Dashed line indicates the lower limit of normal for B cell counts at 80 × 10^6^/mL. (**G**) Proportions of IgD^+^CD27^–^ naive B cells, IgD^–^CD27^+^ mature B cells, and CD38^hi^ plasmablasts within the CD19^+^ B cell compartment in the peripheral blood at day 60. (**H**) Concentration of albumin in the plasma at day 14 in all the participants from the RBWH cohort. Data are presented as the median ± IQR and were analyzed with the Mann-Whitney *U* test (*P < 0.05, **P < 0.01, and ****P < 0.0001).

We recently completed a randomized, placebo-controlled, double-blind phase III clinical trial in which patients undergoing a fully HLA-matched allogeneic BMT were randomized to placebo or tocilizumab on day –1, in addition to standard GVHD prophylaxis with CSA and methotrexate (21). Here, we analyzed data on HCMV reactivation from the Royal Brisbane and Women’s Hospital (RBWH), which accounted for 72% of study participants and where CMV monitoring and treatment were consistent. Patients were monitored for HCMV viremia using a uniform platform and treated with ganciclovir if HCMV viremia levels reached 600 copies/μL on 2 consecutive occasions. None of the patients received HCMV-targeted antiviral prophylaxis. Patients who were serologically positive for HCMV prior to BMT (R^+^) or those receiving grafts from serologically positive donors (D^+^) were considered at risk of HCMV reactivation and included in this analysis. Patient characteristics are presented in Supplemental Table 1. Consistent with data from our earlier phase I/II study, tocilizumab-treated patients had lower rates of HCMV viremia, especially in those receiving an unrelated donor graft (Figure 7A). This association was independent of any effects on acute GVHD and was observed in patients who did not develop significant GVHD (grade 0 or 1 GVHD) (Figure 7B).

Given the importance of recipient-derived humoral immunity in controlling CMV (22) and our current preclinical data, we analyzed the level of HCMV-specific IgG at day 30 after BMT. Both donor and recipient serostatus influenced the level of HCMV IgG (Figure 7C), but the effect of recipient serostatus was dominant. Thus, we limited subsequent analyses to HCMV-seropositive recipients. HCMV IgG levels at day 30 correlated with protection from early HCMV viremia (occurring within the first 5 weeks of BMT), but not viremia that occurred later after BMT (5–14 weeks after BMT), as residual recipient IgG was lost over time (Figure 7D). To our knowledge, these findings provide the first clinical validation of our preclinical studies (22) and suggest there may be different drivers for HCMV viremia at different phases of BMT. Tocilizumab-treated patients had higher levels of HCMV IgG (Figure 7E), and concordant with the HCMV viremia data, the difference was most pronounced in BMT recipients of unrelated donor BM (Figure 7E, right panel). The effect of IL-6 on HCMV IgG levels was not accompanied by any differences in B cell counts at 30 or 60 days after BMT, as the counts remained below normal levels (Figure 7F). Consistent with the limited B cell lineage reconstitution and maturation observed in our experimental models, the reconstituting B cells 2 months after clinical BMT had a predominantly naive (IgD^+^CD27^–^) phenotype, with some IgD^–^CD27^+^ mature B cells and very low numbers of CD38^hi^ plasmablasts (Figure 7G). We also evaluated the effect of IL-6R inhibition on FcRn function by measuring plasma albumin, which is also regulated by FcRn. Consistent with the findings in our preclinical model, tocilizumab-treated patients had significantly higher albumin levels at day 14 (Figure 7H). Collectively, the clinical data confirm our preclinical findings that IL-6 promoted the loss of recipient-derived CMV-specific IgG after BMT and that humoral immunity was critical for reducing HCMV viremia early after transplantation.

## Discussion

Successful outcomes after allogeneic BMT for malignancy are contingent on the elimination of recipient hematopoiesis (and immunity) and replacement by that of donor origin. By its very nature, this process creates a 6- to 12-month window of profound immune suppression, whereby the transplant recipient is at high risk of opportunistic infections, particularly by viral pathogens (6, 32). The development of GVHD generates an additional level of immune deficiency after BMT, both endogenous as a result of inflammation (5, 33), and exogenous as a result of the pharmacological immune suppression that is used to control GVHD (8, 24). CMV reactivation from latency in previously infected individuals remains the most predictable opportunistic infection after BMT. CMV viremia early after transplantation increases the risk of death (2, 3) and also carries a significant economic burden (34). Better treatments for CMV reactivation are essential, and studies that guide the design of improved, safe, and cost-effective therapies that can be rapidly translated into use in immunocompromised transplant recipients are needed. Here, using innovative preclinical models and unique clinical cohorts, we demonstrate that IL-6–dependent inflammation played a major role in the loss of recipient CMV-specific humoral immunity that was critical for the prevention of CMV reactivation early after BMT. IL-6R inhibition had no effect on the development of CMV-specific T cells in either mice or humans. Instead, IL-6 signaling to donor T cells maintained Th1 differentiation and IFN-γ secretion that was critical in mediating endothelial damage, FcRn loss, and the loss of CMV-specific IgG due to impaired recycling. Importantly, the impaired FcRn activity in isolation, either as a result of genetic deletion or pharmacological inhibition, resulted in CMV reactivation, confirming the importance of this pathway in maintaining protective humoral immunity after transplantation,

Humoral immunity, including antibodies and B cells and plasma cells, had been considered largely irrelevant in controlling HCMV after allogeneic BMT (35, 36). Our recent studies revealed that a failure of both cellular and humoral immunity is required for the reactivation of MCMV (22). Although IL-6R inhibition was associated with better recovery of B cells, the reconstituting B cells early after BMT were predominantly of a transitional or naive phenotype and did not contribute to CMV control. Of note, IgG-producing cells (mature B cells and plasma cells) were rapidly eliminated and reconstituted poorly after T cell–replete BMT regardless of IL-6 inhibition and, as such, made a limited contribution to the post-transplantation IgG pool. Critically, we previously demonstrated that MCMV reactivation can be prevented by passively transferred antibodies, with protection being maximal when antibodies are matched to the host MCMV strain (22). The importance of strain-specific antibodies is consistent with the fact that superinfection with multiple genetic variants of HCMV is common in humans (37, 38) and explains the limited success of polyclonal Ig therapy in clinical settings, a finding we recapitulated in our preclinical models (22). Importantly, these data suggest that prolonging the persistence of strain-specific recipient CMV-specific IgG after BMT represents an attractive approach to limit CMV reactivation after BMT. The findings from the current study indicate that IL-6R inhibition assisted in limiting CMV reactivation by prolonging the recipient antibody half-life.

There are 2 principal and not mutually exclusive pathways that may account for the accelerated loss of humoral immunity after BMT: (a) loss of IgG from mucosal sites during GVHD (i.e., protein-losing enteropathy) (39) and (b) enhanced serum IgG catabolism due to defects in the expression of FcRn, which is the Fc receptor responsible for IgG recycling in vivo (15). Here, we define the mechanisms that limit IgG persistence after BMT and the effect of cytokines and GVHD on this process. ECs have the highest levels of functional FcRn and dominate IgG regulation early after allogeneic BMT (15). This is in contrast to FcRn activity at steady state (including late after syngeneic BMT in BM chimeras), in which hematopoietic cells, predominantly macrophages, are the major determinant of FcRn activity (13, 40, 41). This discrepancy reflects the intense proinflammatory state early after allogeneic BMT, which occurs at a time when hematopoietic recovery is limited (42). The increased catabolism or faster loss of IgG after BMT is attributable to liver EC dysfunction, which may be targeted by multiple and redundant pathways. While classical IL-6 signaling aggravates GVHD via an IL-17–dependent mechanism, this pathway does not contribute to the protective effect of T cell–specific IL-6R ablation in relation to CMV reactivation. Instead, our data show that the protection conferred by inhibition of IL-6 signaling involved the inhibition of IFN-γ–dependent, T cell–mediated effects on the endothelium. The finding that IL-6 signaling in T cells was required to maintain Th1 differentiation and IFN-γ secretion after BMT was surprising, since the cytokine is not required to initiate Th1 differentiation (18). Thus, agents that block the initiation of Th1 differentiation, for example, the inhibition of IL-12 with ustekinumab, may be effective, and these data will be available in the next 2 years (ClinicalTrials.gov, NCT04572815). The JAK1/2 inhibitor ruxolitinib represents another promising agent, since it is effective in attenuating cytokine signaling during GVHD (43), but the effects of this agent on CMV reactivation will require prospective randomized studies using it as GVHD prophylaxis. Furthermore, the deletion of IL-6 and IFN-γ from our transplant systems did not completely rescue ECs from injury, and we speculate that other pathways such as type I IFNs (44) and perforin-granzyme–mediated cytolysis may be additionally involved.

Our findings are consistent with the notion that FcRn expression and the associated effects on IgG pharmacokinetics are mediated by cytokine-dependent effects on ECs and are not directly dependent on the severity of GVHD per se. Thus, IL-6 and IFN-γ demonstrated multifaceted effects during the pathogenesis of GVHD and represented key mediators of EC injury that were not tractable by calcineurin inhibition–based immune suppression (Supplemental Figure 7).

In sum, CMV-specific humoral immunity of recipient origin plays a critical role in preventing CMV reactivation early after BMT, until such time as an effective donor T cell response can be generated. This protective recipient-derived humoral immunity is rapidly lost as a consequence of endothelial damage after BMT, a process that is IL-6 and IFN-γ dependent and predisposes the patient to CMV reactivation. Cytokine inhibition thus represents an attractive therapeutic approach to maintain virus-specific humoral immunity in disease settings characterized by high states of inflammation. Finally, these data suggest that approaches to prevent endothelial injury after BMT may have important effects on the maintenance of humoral immunity.

## Methods

### Sex as a biological variant.

The preclinical murine systems utilized female mice. There is no clinical evidence to suggest that CMV reactivation after BMT is sex restricted, and our clinical studies enrolled both male and female recipients.

### Mice.

Female C57BL/6 (H-2b), B6D2F1 (H-2b/d), and BALB/c mice were purchased from the Animal Resources Centre (Perth, Western Australia, Australia), The Jackson Laboratory, or were bred in the animal facilities of the Fred Hutchinson Cancer Center. *Cd4^Cre^* *Il6r^fl/fl^*, μMt, *Il17a^–/–^*, and TNFR1/2-dKO (*Tnfr1/2^–/–^*) mice were on a C57BL/6J (B6) background and bred at the QIMR Berghofer (Brisbane, Queensland, Australia) or the Fred Hutchinson Cancer Center. *Fcrn^–/–^*, *Ifng^–/–^*, *Tnf^–/–^*, and age-matched C57BL/6J mice were purchased from The Jackson Laboratory. Donor mice were age-matched females, and recipient mice were used between 8 and 10 weeks of age. To generate MCMV latency, female mice at 6–8 weeks of age were infected i.p. with 1 × 10^4^ PFU salivary gland–propagated MCMV-K181^Perth^ (K181) and rested for more than 90 days (22). Mice were housed in microisolator cages and received acidified autoclaved water (pH 2.5) after BMT. Sample sizes were determined on the basis of initial and previous results to ensure appropriate power (*n* values in the figure legends represent individual mice).

### BMT.

BMT was performed as described previously (45). Briefly, mice received 1,100 (B6D2F1) or 1,000 (C57BL/6) cGy total body irradiation (^137^Cs source at 108 cGy/min) on day –1 and were administered BM and T cells (BM+T groups) or TCD BM (non-GVHD control group) on day 0. T cells were purified from spleens using magnetic bead depletion (46) with CD3^+^ T cell purities of greater than 80% containing both CD4^+^ and CD8^+^ T cells. TCD BM was prepared using a complement-mediated pan–T cell depletion method (47), resulting in greater than 90% depletion. GVHD was scored with a clinical scoring system as previously described (45). Briefly, mice were monitored regularly using the following 5 clinical parameters: weight loss, posture (hunching), activity, fur texture, and skin integrity (2 for each parameter, with a maximum index of 10) and sacrificed if their clinical score was 6 or higher. For animals receiving GVHD prophylaxis, CSA (5 mg/kg/d) was administered i.p. from day 0 to day 13 after BMT.

### MCMV quantification.

Quantification of the MCMV viral load was performed as previously described (22). In brief, MCMV glycoprotein B (gB) DNA in plasma was determined by real-time quantitative PCR (qPCR) and a SYBR Green assay (Bio-Rad) using gB-specific primers (forward: 5′-TTGGCTGTCGTCTAGCTGTTT-3′ and reverse: 5′-TAAGGCGTGGACTAGCGATAA-3′). The standard curve was generated by serial dilution of a synthesized MCMV gB sequence with a limit of detection of 4 copies/μL plasma. The viral load in target organs was determined by plaque-forming assay, with a limit of detection of 40 PFU per organ.

### Detection of MCMV-specific T cells.

Virus-specific CD8^+^ T cells were quantified by flow cytometry using m38 tetramers as previously described (22). PE-conjugated m38 tetramers (H-2Kb-SSPPMFRV) were from Immuno ID Tetramers (Melbourne, Victoria, Australia) or the NIH tetramer core facility at Emory University (Atlanta, Georgia, USA). Tetramers were titrated and used at the optimum dilution.

### Quantification of MCMV-specific Ig.

MCMV-specific IgG was quantified by ELISA (22). Briefly, MCMV antigen-precoated 96-well plates were incubated with serum samples for 2 hours and washed before subsequent incubation with anti–mouse IgG (GE Healthcare) or IgM (SouthernBiotech) peroxidase conjugates for 1 hour. The plates were then washed and incubated with tetramethylbenzidine substrate (ELISA Systems) for 5–20 minutes at room temperature. The reaction was terminated by the addition of 1 M sulfuric acid followed by reading on an Epoch plate reader (BioTek) at 450 nm. Serum from uninfected mice was used as a negative control.

### Pharmacokinetics analysis of administered IgG2b.

A monoclonal mouse IgG2b specific for human CD4 (does not cross-react with mouse CD4) was administered i.v., and the plasma levels were monitored by flow cytometry. The sample process and flow cytometric analysis were conducted as previously described with modifications (28). Briefly, mouse anti–human CD4 antibody (IgG2b, clone OKT4, Abcam) or test samples were diluted in PBS plus 2% BSA and incubated with freshly isolated human PBMCs for 30 minutes at 4°C. The PBMCs were subsequently stained with rat anti–mouse IgG2b phycoerythrin (PE), followed by staining with anti–human CD3 and anti–human CD4 antibodies before analysis on a flow cytometer. The IgG2b concentration in test samples correlated with the intensity of the PE signal on CD3^+^CD4^+^ T cells and was measured using standard curves. The concentration of administered IgG2b followed 2-phase decay kinetics, whereby the half-life was calculated from the second phase.

### Isolation of ECs and hematopoietic cells from liver.

Isolation of ECs from liver was conducted as described previously (29) with modifications. Briefly, 5 U heparin was i.p. administered to mice at least 5 minutes before sacrifice. Following the sacrifice of mice, the liver was perfused with PBS and cut into small pieces with scissors after removal of the gallbladder. The tissues were serially digested with (a) dispase II (1 U/mL, MilliporeSigma) plus DNase I (10 μg/mL, MilliporeSigma), (b) type I collagenase (1 mg/mL, Life technologies, Thermo Fisher Scientific), and (c) type II collagenase (1 mg/mL, MilliporeSigma) in HBSS plus 4% FCS at 37°C for 15 minutes. CaCl_2_ (900 μM) and MgCl_2_ (330 μM) were supplemented to the collagenases. Digested tissues were broken into single-cell suspensions by passing through 18 gauge needles 7–8 times, lysed for RBCs, and resuspended in IMDM complete medium. Tissues or cell suspensions were centrifuged at 330*g* for 3 minutes to remove supernatant. The cell suspensions contained a mixture of ECs and hematopoietic cells.

### FcRn-mediated IgG uptake assay and in vivo IgG depletion assay.

FcRn-mediated IgG uptake was determined as previously described (13). In brief, single-cell suspensions were incubated with FcγRIIB/III antibody (2.4G2) at 4°C for 30 minutes, washed, and then incubated with Alexa 647–labeled MST-HN or H435A mutated human IgG variants at 37°C for 45 minutes in phenol red–free DMEM plus 10% IgG-depleted FCS. The cells were then washed, surface stained, and analyzed by flow cytometry. MST-HN is a derivative of human IgG1 with mutations in Met252, Ser254, Thr256, His433, and Asn434, which binds FcRn with increased affinity and reduced pH dependence (30). H435A is a derivative of human IgG1 with negligible binding to FcRn. Thus, MST-HN staining is indicative of the levels of functional FcRn, and H435A staining represents nonspecific, fluid-phase uptake. The geometric MFI of MST-HN is used to represent the level of functional FcRn. For in vivo depletion of IgG after BMT, an in-house–produced MST-HN derivative (MG14, Fc fraction, biosimilar to efgartigimod) was administered i.v. in 2 doses (350 μg on day 0 and 150 μg on day 3); a derivative of unmutated IgG1 (MG12) was used as a control. Expression constructs to produce WT human IgG1–derived Fc and the mutated variant MST-HN (with “Abdeg” mutations: M252Y/S254T/T256E/H433K/N434F; refs. 30, 48), comprising the mouse Ig leader peptide (MAVLVLFLCLVAFPSCVLS) and human Fc genes (starting at residue D221, EU numbering), were generated using standard methods of molecular biology. The genes were assembled into the expression vector pcDNA3.4 (Thermo Fisher Scientific). Recombinant Fc fragments were expressed by transiently transfected HEK293 cells (Expi293FTM, Gibco, Thermo Fisher Scientific) using the Expifectamine Transfection Kit (Gibco, Thermo Fisher Scientific) and purified from culture supernatants using protein A–sepharose. Aggregates were removed using size exclusion chromatography (Yarra SEC-3000 3 μm GFC column, Phenomenex). Sequences of the expressed proteins are listed in Supplemental Table 2 (MG14) and Supplemental Table 3 (MG12).

### Endocytosis assay.

pHrodo green dextran (molecular weight, 10,000 MW; Invitrogen, Thermo Fisher Scientific) contains pH-sensitive fluorescent dye that is nearly nonfluorescent in the neutral extracellular environment and increases in fluorescence in the acidic environment (pH <6) within endosomes and lysosomes. The assay was conducted according to the manufacturer’s protocol. Briefly, the single-cell suspension was blocked with 2.4G2, stained for surface markers, washed, incubated with pHrodo green dextran (40 μg/mL) at 37°C for 20 minutes in IMDM complete medium, and then kept on ice before analysis on a flow cytometer. Dead cells were excluded with 7-AAD.

### Single-cell RNA-Seq and data analysis.

Livers were taken on day 7 after murine BMT, digested, pooled from each group (*n =* 3 per group), sort purified to CD31^+^CD45^–^TER119^–^ ECs, and subsequently captured on a 10X Genomics Chromium platform with the 5-prime kit. Libraries were sequenced on the NextSeq 2000 (Illumina P3 kit) targeting 20,000 reads per cell. Illumina BCL reads were demultiplexed and prepared using Cell Ranger Multi (10X Genomics) and filtered using the following parameters: 3 <log RNA <5; percentage of mitochondrial RNA <15%. Doublets were identified using Scrublet, and cells with a doublet score above 0.2 were removed. RNA counts were normalized using SCT transform (49). Cell clusters were identified using a standard Seurat workflow. Differentially expressed genes across experimental groups were used as input to fgsea (50) for gene set enrichment. Hallmark pathway gene set terms were sourced from MSigdb. SCpubr (51) and decoupleR (52) were used to infer pathway activities across groups.

### Clinical studies.

Patients were enrolled in 1 of the following 3 prospective clinical studies: (a) an observational study entitled “Observational study of IL-17–related cytokines in stem cell transplantation” (HREC/15/QRBW/299) (53), which was also used as the control arm for (b) a phase I/II study of tocilizumab to prevent the development of acute GVHD after HLA-matched allogeneic hematopoietic progenitor cell transplantation (HREC/11/QRBW/345, anzctr.org.au study number: ACTRN12612000726853) (19), and (c) a phase III randomized, double-blind, placebo-controlled study of tocilizumab to prevent the development of acute GVHD after HLA-matched allogeneic hematopoietic progenitor cell transplantation (HREC/14/QRBW/35, anzctr.org.au study number ACTRN12614000266662) (21). HCMV seropositivity in the donor and/or recipient was used to define at-risk patients. Patients were excluded for analysis if both donor and recipient were HCMV-seronegative, had CMV reactivation prior to day 0 or died (or were lost during follow-up) before day 100 before reactivation.

### HCMV viremia.

HCMV viral load was monitored using the COBAS Amplicator CMV Monitor test (Roche Diagnostics) as previously described (54). The cumulative incidence of HCMV viremia at any level above the limit of detection was analyzed up to day 100 after BMT. Patients were censored at the time of death.

### HCMV-specific IgG.

HCMV IgG was quantified using a chemiluminescence method on the Diasorin Liaison XL platform by Sullivan Nicolaides Pathology. This semiquantitative method had a detection range of 5–180 U/mL.

### Flow cytometry.

Antibody-stained single-cell suspensions were analyzed on an LSR Fortessa cytometer in Australia or a Symphony A3 in Seattle, Washington, USA (both from BD Biosciences), and data were processed using FlowJo, version 10 (BD Life Sciences).

### Statistics.

Results are presented as the median ± IQR. The Mann-Whitney *U* test was used for comparison between 2 groups, and the Kruskal-Wallis test was used for multiple comparisons. The χ^2^ or Fisher’s exact test was used for comparison of categorical variables. The ordinary least-squares method was used in linear or semi-log regression analyses. A 2-sided *P* value of 0.05 was considered statistically significant. Statistical analyses were performed using GraphPad Prism, version 8 (GraphPad Software). The cumulative incidence for HCMV reactivation was estimated and plotted using cmprsk package R, version 3.5.3.

### Study approval.

All animal studies were approved by the animal ethics committees at QIMR Berghofer, the Lions Eye Institute, and the IACUC of the Fred Hutchinson Cancer Center. All clinical studies were approved by the Royal Brisbane and Women’s Hospital Human Research Ethics Committee, and all patients provided informed consent.

### Data availability.

RNA-Seq data have been deposited in the NCBI’s Gene Expression Omnibus (GEO) database and are publicly available as of the date of publication (GEO GSE252180). Original data are available in the Supplemental Supporting Data Values file.

## Author contributions

PZ designed and performed murine experiments, analyzed murine and human data, and wrote the manuscript. PF and CEA designed and performed some murine experiments, analyzed the relevant data, and critically edited the manuscript. OGW, SSB and SNF analyzed RNA-Seq data. JPM, BAM, VV, SD, RDK, APE, AS, SO, AV, CS, CRS, KSE, SRWL, TS, SAM, and MK helped perform research. MG and IW manufactured the FcRn reagents. ADC reviewed and scored the histopathological images. ASH and GAK helped collect clinical data. ESW provided critical reagents and expertise in FcRn biology and edited the manuscript. MADE conceptualized and designed the study, supervised the research, and wrote the manuscript. GRH conceptualized the study, supervised the research, and wrote the manuscript. SKT collected clinical data, conceptualized the study, supervised the research, and wrote the manuscript.

## Supplementary Material

Supplemental data

Supporting data values

## Version 1 04/01/2024

Electronic publication
