# Supplementary material for: IL-6–mediated endothelial injury impairs antiviral humoral immunity after bone marrow transplantation
Source: J Clin Invest. 2024 Apr 1;134(7):e174184. doi: 10.1172/JCI174184 (PMC10977988; doi:10.1172/JCI174184)
Supplement: Supplemental data [file jci-134-174184-s015.pdf]

Supplementary Methods and Materials

Methods

Detection of MCMV-specific CD4$^+$ T cells. For the detection of virus-specific CD4$^+$ T cells, BM-derived DC (C57BL/6) were incubated with MCMV (K181) overnight before co-culture with splenocytes at responder:stimulator ratio of 5:1 for 4 hours in the presence of brefeldin A (1), following which the cells were stained for intracellular cytokines (IFNγ and TNF) and analyzed by flow cytometry.

Gene expression analysis. Splenic CD4$^+$ T cells were FACS-sorted at day +21 after BMT and total RNA extracted with the RNeasy Micro Kit (Qiagen). Gene expression of T_{FH}-related markers was determined using the TaqMan gene expression assay (Applied Biosystems) and normalized to the house-keeping gene, *Hprt*.

Isolation of liver mononuclear cells. In some experiments (where indicated in figure legends), Mononuclear cells were isolated from liver using Percoll gradient centrifugation method as previously described (2).

Plasma cytokine levels. Cytokine levels were determined using the BD Cytometric Bead Array system (BD Biosciences).

Histology. Histology of GVHD target organs was performed as described before (3). Briefly, formalin-fixed tissues were embedded in paraffin, processed to 5µm sections, H&E stained and examined in a blinded fashion (by A.D.C). Images were scanned at 20X magnification using an Aperio AT Turbo slide scanner (Leica Biosystems, Wetzlar, Germany) and analyzed using Aperio ImageScope software (Leica).

References

1. Andrews DM, Estcourt MJ, Andoniou CE, Wikstrom ME, Khong A, Voigt V, et al. Innate immunity defines the capacity of antiviral T cells to limit persistent infection. *J Exp Med.* 2010;207(6):1333-43.
2. Zhang P, Lee JS, Gartlan KH, Schuster IS, Comerford I, Varelias A, et al. Eomesodermin promotes the development of type 1 regulatory T (T(R)1) cells. *Sci Immunol.* 2017;2(10).
3. Burman AC, Banovic T, Kuns RD, Clouston AD, Stanley AC, Morris ES, et al. IFNgamma differentially controls the development of idiopathic pneumonia syndrome and GVHD of the gastrointestinal tract. *Blood.* 2007;110(3):1064-72.

Mouse Antibodies.

Antibody	Clone	Fluorochromes	Catalog #	Suppliers
CD3	145-2C11	PE/Cy7	100320	Biolegend
CD3	145-2C11	PE/Cy5	100310	Biolegend
CD90.2 (Thy1.2)	53-2.1	BV605	140318	Biolegend
CD90.2 (Thy1.2)	53-2.1	BV480	566075	BD Bioscience
CD4	GK1.5	AF700	100430	Biolegend
CD4	GK1.5	BUV496	612952	BD Bioscience
CD4	GK1.5	BV786	100453	Biolegend
CXCR-5 (CD185)	L138D7	PE	145504	Biolegend
PD-1 (CD279)	J43	BUV737	568362	BD Bioscience
PD-1 (CD279)	RMPI-30	PE/Cy7	109110	Biolegend
CD44	IM7	APC/Cy7	103028	Biolegend
CD8	53-6.7	PerCP/Cy5.5	100710	Biolegend
CD8	53-6.7	APC/Cy7	100714	Biolegend
CD8	53-6.7	BUV805	612898	BD Bioscience
CD45.1	A20	APC/Cy7	110716	Biolegend
CD45.1	A20	BUV395	565212	BD Bioscience
CD45.2	104	FITC	109806	Biolegend
CD45.2	104	AF700	109822	Biolegend
H2Dd	34-2-12	FITC	110606	Biolegend
H2Dd-biotin	34-2-12	N/A	110606	Biolegend
H2Db	KH95	PE	111508	Biolegend
CD19	1D3	BV786	563333	BD Bioscience
B220 (CD45R)	RA3-6B2	BUV496	612950	BD Bioscience
IgM	RMM-1	PE/Cy7	406514	Biolegend
IgD	11-26c.2a	APC/Cy7	405716	Biolegend
GL-7	GL-7	FITC	144614	Biolegend
Fas (CD95)	SA367H8	APC	152604	Biolegend
CD138	281-2	BV421	142507	Biolegend
CD11b	M1/70	PerCP/Cy5.5	101228	Biolegend
F4/80	BM8	PE	123110	Biolegend
F4/80	BM8	AF700	123130	Biolegend
CD11c	N418	BV421	117330	Biolegend
CD45	30-F11	BUV395	564279	BD Bioscience
CD31	MEC13.3	PE/Cy7	102524	Biolegend
VCAM-1 (CD106)	429 (MVCAM.A)	PE	105714	Biolegend
IA/IE	M5/114.15.2	BV510	107636	Biolegend
TER119	TER-119	BV605	116239	Biolegend
IFNγ	XMG1.2	BV421	505830	Biolegend
IFNγ	XMG1.2	BV785	505838	Biolegend
TNF	MP6-XT22	BB700	566510	BD Bioscience
T-bet	4-B10	AF647	644804	Biolegend
Rat-anti-mouse IgG2b	RMG2b-1	PE	406708	Biolegend
pHrodo Green Dextran	N/A	N/A	P35368	Life technologies
CD16/CD32 (Fc Block)	2.4G2	N/A	553142	BD Bioscience

Human antibodies and other reagents

Antibody	Clone	Fluorochromes	Catalog #	Supplier
CD19	HIB19	FITC	302206	Biolegend
HLA-DR	L243	PE/Cy7	307616	Biolegend
CD38	HIT2	APC/Cy7	303534	Biolegend
CD27	L128	BV605	562655	BD Bioscience
IgD	IA6-2	AF700	348229	Biolegend
7-Aminoactinomycin D	N/A	N/A	SML1633	Sigma-Aldrich
Fixable Viability Stain 440UV	N/A	N/A	566332	BD Bioscience
Zombie aqua dye	N/A	N/A	77143	Biolegend

Table S1. Baseline characteristics of patients

	Placebo	Tocilizumab
Gender		
Male	29 (62%)	20 (53%)
Female	18 (38%)	18 (47%)
Age	52 (19 – 68)	55 (20 – 68)
Conditioning		
Myeloablative	19 (40%)	13 (34%)
Reduced intensity	28 (60%)	25 (66%)
CMV serostatus		
D-R+	16 (34%)	11 (29%)
D+R+	24 (51%)	18 (47%)
D+R-	7 (15%)	9 (24%)
Donors		
Sibling	19 (40%)	12 (32%)
Unrelated donor	28 (60%)	26 (68%)
Acute GVHD		
0 – I	28 (60%)	27 (71%)
II – IV	19 (40%)	11 (29%)

Race and ethnicity data are not available.

Table S2: Construct map of human IgG Fc with mutations ‘KF’ in CH3 and ‘YTE’ in CH2 (MG14).

Nucleotide sequence with highlighted domains<br><br>ATGGCTGTTCTGGTGCTGTTCCTCTGCCTGGTTGCATTTCCAAGCTGTGTCCTGTCCGACAAAACTC<br>ACACA*TGCCCACCGTGCCCA*GCACCTGAACTCCTGGGGGGACCGTCAGTCTTCCTCTTCCCCCCAA<br>AACCCAAGGACACCCTC**TAT**ATC**ACC**CGG**GAA**CCTGAGGTCACATGCGTGGTGGTGGACGTGAGC<br>CACGAAGACCCTGAGGTCAAGTTCAACTGGTACGTGGACGGCGTGGAGGTGCATAATGCCAAGAC<br>AAAGCCGCGGGAGGAGCAGTACAACAGCACGTACCGTGTGGTCAGCGTCCTCACCGTCCTGCACC<br>AGGACTGGCTGAATGGCAAGGAGTACAAGTGCAAGGTCTCCAACAAAGCCCTCCCAGCCCCCATC<br>GAGAAAACCATCTCCAAAGCCAAAGGGCAGCCCCGAGAACCACAGGTGTACACCCTGCCCCCATC<br>CCGGGATGAGCTGACCAAGAACCAGGTCAGCCTGACCTGCCTGGTCAAAGGCTTCTATCCCAGCG<br>ACATCGCCGTGGAGTGGGAGAGCAATGGGCAGCCGGAGAACAACTACAAGACCACGCCTCCCGTG<br>CTGGACTCCGACGGCTCCTTCTTCCTCTACAGCAAGCTCACCGTGGACAAGAGCAGGTGGCAGCA<br>GGGGAACGTCTTCTCATGCTCCGTGATGCATGAGGCTCTG**AAGTTC**CACTACACGCAGAAGAGCCT<br>CTCCCTGTCTCCGGGTAAATAA
Amino-acid sequence with highlighted domains<br><br>MAVLVLFLCLVAFPSCVLSDKTHTCPPCPAPELLGGPSVFLFPPKPKDTL**Y**I**T**R**E**PEVTCVVVDVSHEDPE<br>VKFNWYVDGVEVHNAKTKPREEQYNSTYRVVSVLTVLHQDWLNGKEYKCKVSNKALPAPIEKTISKAKG<br>QPREPQVYTLPPSRDELTKNQVSLTCLVKGFYPSDIAVEWESNGQPENNYKTTPPVLDSDGSFFLYSKL<br>TVDKSRWQQGNVFSCSVMHEAL**KF**HYTQKSLSLSPGK

Notes: Signal Hinge CH2 CH3

Table S3. Construct map of human IgG Fc wild type (MG12)

Nucleotide sequence with highlighted domains
ATGGCTGTTCTGGTGCTGTTCCTCTGCCTGGTTGCATTTCCAAGCTGTGTCCTGTCCGACAAAACTC<br>ACACATGCCCACCGTGCCCAGCACCTGAACTCCTGGGGGGACCGTCAGTCTTCCTCTTCCCCCCAA<br>AACCCAAGGACACCCTCATGATCTCCCGGACCCCTGAGGTCACATGCGTGGTGGTGGACGTGAGC<br>CACGAAGACCCTGAGGTCAAGTTCAACTGGTACGTGGACGGCGTGGAGGTGCATAATGCCAAGAC<br>AAAGCCGCGGGAGGAGCAGTACAACAGCACGTACCGTGTGGTCAGCGTCCTCACCGTCCTGCACC<br>AGGACTGGCTGAATGGCAAGGAGTACAAGTGCAAGGTCTCCAACAAAGCCCTCCCAGCCCCCATC<br>GAGAAAACCATCTCCAAAGCCAAAGGGCAGCCCCGAGAACCACAGGTGTACACCCTGCCCCCATC<br>CCGGGATGAGCTGACCAAGAACCAGGTCAGCCTGACCTGCCTGGTCAAAGGCTTCTATCCCAGCG<br>ACATCGCCGTGGAGTGGGAGAGCAATGGGCAGCCGGAGAACAACTACAAGACCACGCCTCCCGTG<br>CTGGACTCCGACGGCTCCTTCTTCCTCTACAGCAAGCTCACCGTGGACAAGAGCAGGTGGCAGCA<br>GGGGAACGTCTTCTCATGCTCCGTGATGCATGAGGCTCTGCACAACCACTACACGCAGAAGAGCCT<br>CTCCCTGTCTCCGGGTAAATAA
Amino-acid sequence with highlighted domains
MAVLVLFLCLVAFPSCVLSDKTHTCPPCPAPELLGGPSVFLFPPKPKDTLMISRTPEVTCVVVDVSHEDP<br>EVKFNWYVDGVEVHNAKTKPREEQYNSTYRVVSVLTVLHQDWLNGKEYKCKVSNKALPAPIEKTISKAK<br>GQPREPQVYTLPPSRDELTKNQVSLTCLVKGFYPSDIAVEWESNGQPENNYKTTPPVLDSDGSFFLYSK<br>LTVDKSRWQQGNVFSCSVMHEALHNHYTQKSLSLSPGK

Notes: Signal Hinge CH2 CH3

Figure S1. Effects of T-cell specific ablation of IL-6R on the development of MCMV-specific T cell responses

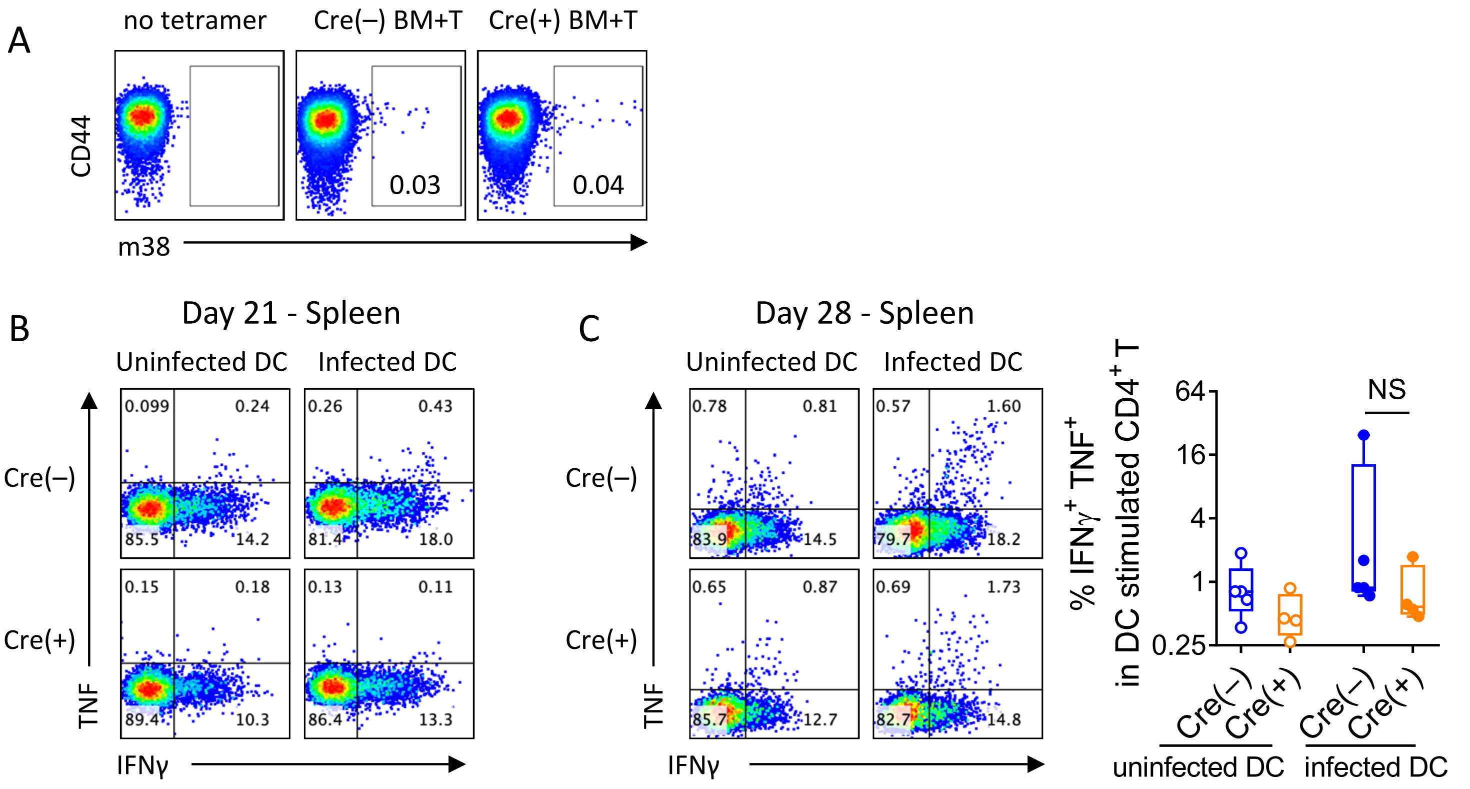

Latently infected B6D2F1 recipients were transplanted with BM (5x10^{6}) and CD3^{+} T cells (2x10^{6}) from B6.*Cd4*$^{cre+}$ x *Il6r*$^{fl/fl}$ mice (Cre+) and their littermate controls (Cre^{-}). (A) Flow cytometric plots of m38 tetramer staining on splenic CD8^{+} T cells at 2 weeks after BMT. Plots were concatenated from 5 samples each and shown is a representative example from 2 experiments. (B – C) Spleens were stimulated with MCMV-infected DC *in vitro* to quantify MCMV-specific CD4^{+} T cell responses. Expression of IFNγ and TNF in splenic CD4^{+} T cells at (B) day 21 (n = 3 per group) and (C) day 28 (n = 4 – 5 per group) after BMT. Data are presented as median ± interquartile range and was analyzed with the Mann-Whitney U test. NS, not significant.

Figure S2. Effects of T-cell specific ablation of IL-6R on humoral immunity after BMT

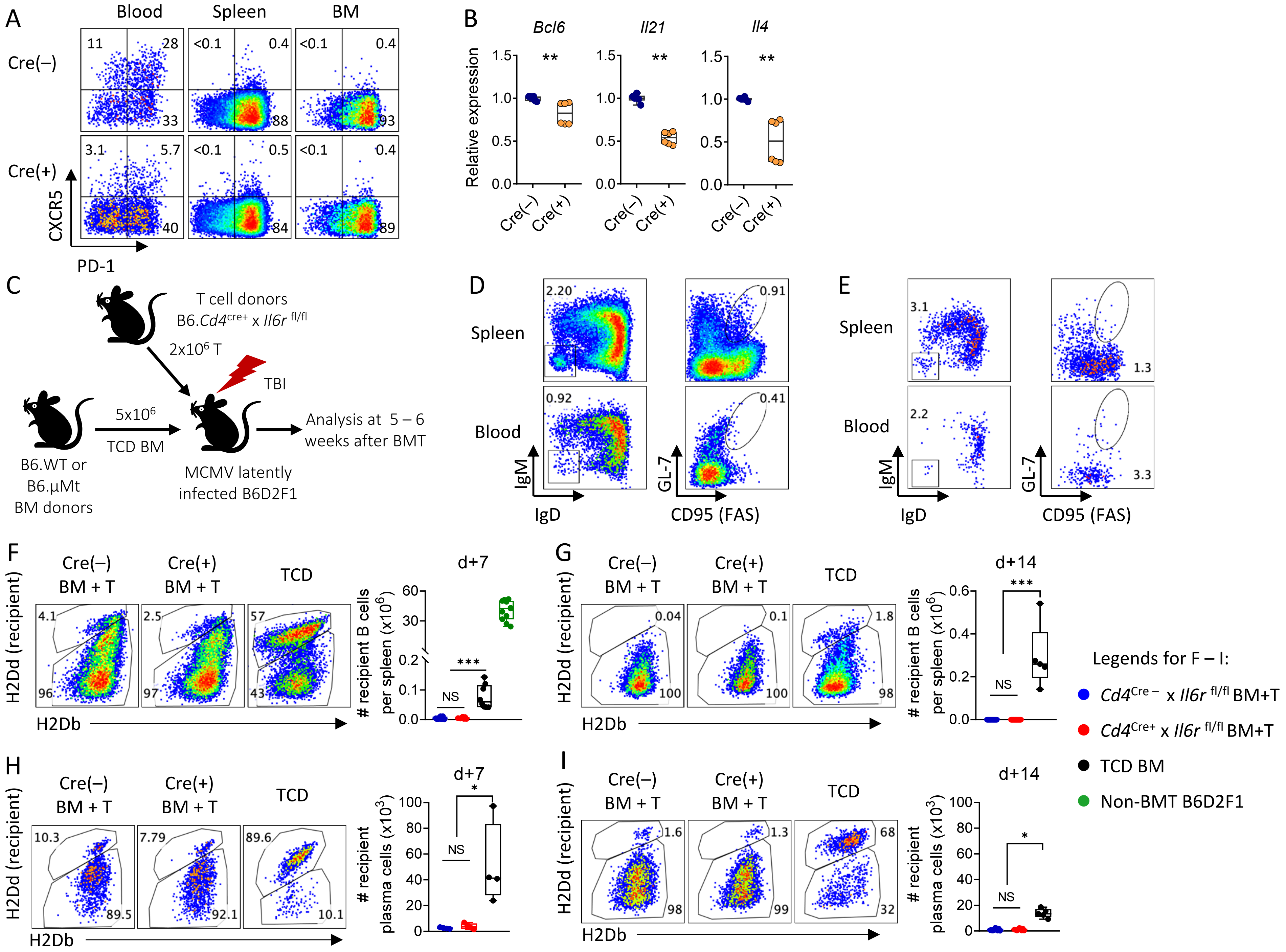

Figure S2. Effects of T-cell specific ablation of IL-6R on humoral immunity after BMT

(A – B) B6D2F1 recipients were transplanted with BM ($5x10^6$) and T cells ($2x10^6$) from B6.*Cd4*$^{cre+}$ x *Il6r*$^{fl/fl}$ mice (Cre+) or littermate controls (Cre$^-$). (A) Flow cytometric plots showing PD-1$^+$CXCR5$^+$ follicular helper CD4$^+$ T (T_{FH}) cells in the blood, spleen and BM. Plots were concatenated from 3 – 4 samples each and shown is a representative example from 2 experiments. (B) Relative expression of T_{FH}-related markers as determined by RT-PCR on FACS-sorted splenic CD4$^+$ T cells. Each dot represents individual mice (*n* = 6 per group from 2 experiments). (C – D) Latently infected B6D2F1 recipients were transplanted with BM ($5x10^6$) from C57BL/6J (WT) or B6.µMt mice and T cells ($2x10^6$) from B6.*Cd4*$^{cre+}$ x *Il6r*$^{fl/fl}$ mice (Cre+). (C) Experimental schema. (D) Representative flow cytometric plots of splenic and blood B cells (gated on CD90.2$^-$ CD11b$^-$CD19$^+$CD138^{dim} B cells) in recipients of WT BM at 6 weeks after BMT showing low frequencies of class-switched (IgM$^-$IgD$^-$) and germinal center (GL7$^+$FAS$^+$) B cells. Plots were concatenated from 5 samples and shown is a representative example from 2 experiments. (E) Latently infected B6D2F1 recipients were transplanted with BM ($5x10^6$) and T cells ($2x10^6$) from B6.*Cd4*$^{cre-}$ x *Il6r*$^{fl/fl}$ mice (Cre$^-$ or WT). Representative flow cytometric plots of splenic and blood B cells at 5 weeks after BMT show the frequencies of class-switched (IgM$^-$IgD$^-$) and germinal center (GL7$^+$FAS$^+$) B cells. Plots were concatenated from 4 – 5 samples and shown is a representative example from 2 experiments. (F – I) B6D2F1 recipients were transplanted with BM ($5x10^6$) and T cells ($2x10^6$) from B6.*Cd4*$^{cre+}$ x *Il6r*$^{fl/fl}$ mice (Cre+) or littermate controls (Cre$^-$) and spleens were taken for analysis on days +7 and +14 after BMT. Donor/recipient chimerism was determined by expression of H2Db and H2Dd using flow cytometry. Representative flow cytometric plots are concatenated from 4 – 5 samples per group. Recipient CD19+ B cells in the spleens on day +7 (F, n = 8 – 11 per group from 2 experiments) and day +14 (G, GVHD groups: n = 12 – 15 from 2 experiments, TCD group: n = 5 from 1 experiment). Recipient CD19$^-$ CD138$^+$ plasma cells in the spleens on day +7 (H, n = 4 per group from 1 experiment) and day +14 (I, n = 5 – 9 per group from 1 experiment). BM + T indicates bone marrow plus T cells. Data are presented as median ± interquartile range and was analyzed with the Mann-Whitney U test. *$P < 0.05$, **$P < 0.01$.

Figure S3. Measurement of IgG clearance after BMT

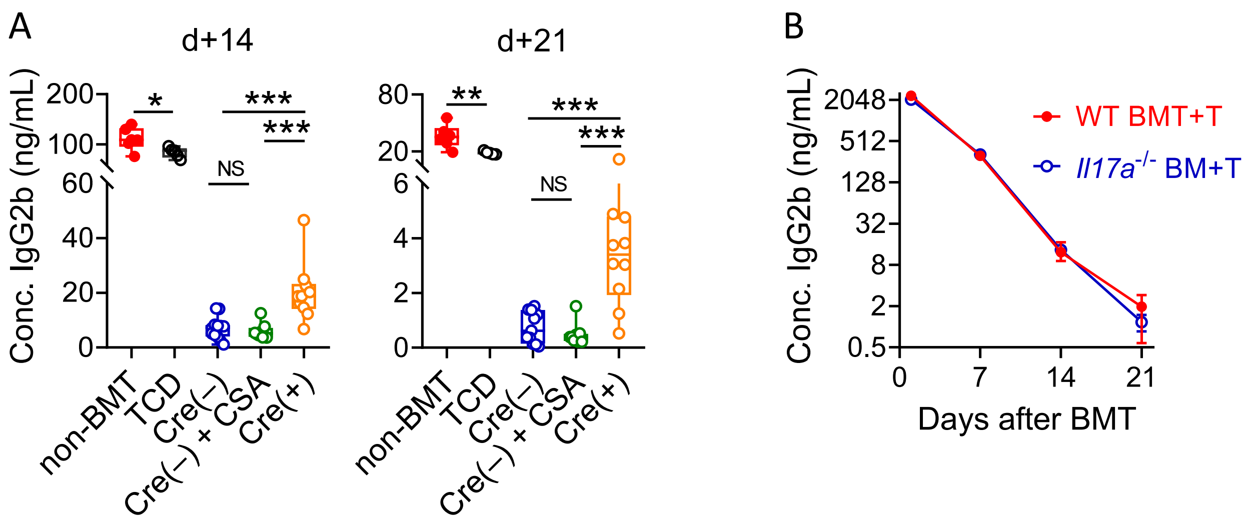

(A) Experiments were conducted as described in Figure 3G. Concentration of exogenously administered mouse IgG2b was determined on days +14 and +21 after BMT and shown by treatment groups; the data are the same as those in Figure 3G but individual mice are shown. (B) B6D2F1 recipients were transplanted with BM ($5x10^6$) and T cells ($2x10^6$) from C57BL/6J or *Il17a*$^{-/-}$ donors (n = 8 per group from 1 experiment). Concentration of exogenously administered mouse IgG2b (administered intravenously on day 0 together with graft) in the plasma is plotted. Data are presented as median ± interquartile range and was analyzed with the Mann-Whitney U test. NS, not significant; *P < 0.05; **P < 0.01; ***P < 0.001.

Figure S4. Role of hematopoietic cells and endothelial cells in IgG recycling after BMT

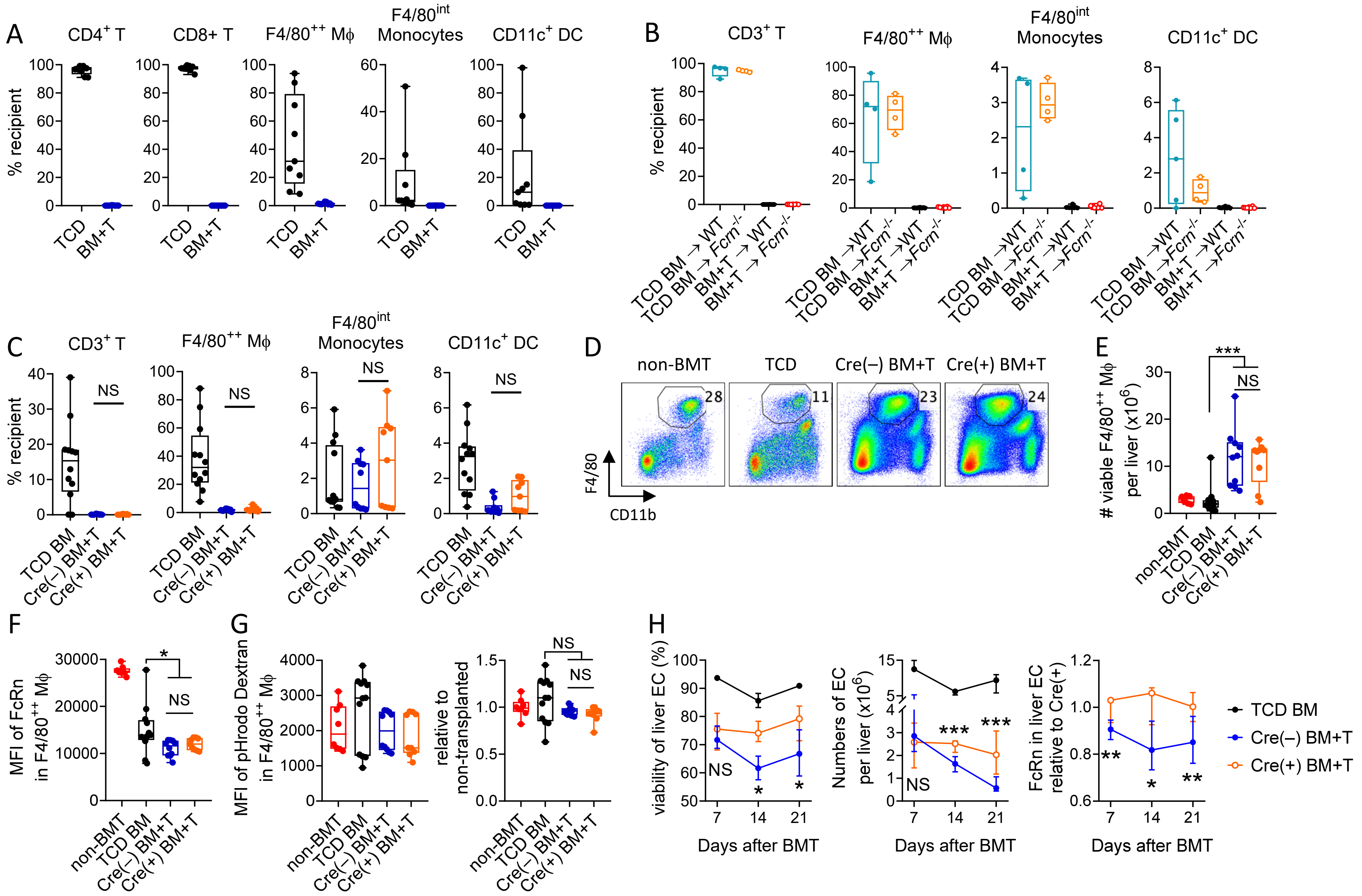

Figure S4. Role of Hematopoietic cells and endothelial cells in IgG recycling after BMT

(A and B) BM ($10x10^6$) ± T cells ($3 – 5 x10^6$) from BALB/c donors were transplanted into non-infected B6 recipients. TCD represents BM without T cells. Liver cells were isolated on day +14 (A, *n* = 9 per group from 2 experiments) or +21 (B, *n* = 4 – 8 per group from 1 experiment) and analyzed for donor/recipient chimerism in various cell subsets by staining the expression of $H2D^b$ (recipient) and $H2D^d$ (donor). (C – G) BM ($5x10^6$) ± T cells ($2x10^6$) from *Cd4*$^{cre+}$ x *Il6r*$^{fl/fl}$ donors (Cre+) and littermate controls (Cre–) were transplanted into non-infected B6D2F1 recipients. Liver cells were isolated on day +14 (*n* = 8 – 12 per group from 2 experiments). (C) Donor/recipient chimerism in various cell subsets by staining for the expression of $H2D^b$ (donor) and $H2D^d$ (recipient); (D) Representative flow cytometric plots showing frequencies of F4/80^{++} macrophages (Mϕ) in CD45$^+$CD31$^-$gate; (E) numbers of viable F4/80^{++} macrophages per liver; (F) MFI of FcRn in viable F4/80^{++} macrophages; (G) MFI of pHrodo Dextran in viable F4/80^{++} macrophages (left panel) with normalized expression relative to the mean value of non-BMT group (right panel). (H) Transplantation was set up as described in (C – G). Liver ECs were isolated on days +7, +14 and +21 and analyzed for viability, absolute numbers and FcRn expression (n = 8 – 12 per group per time point from 2 experiments; the day +14 timepoint data are also presented in Figure 4). The statistical comparisons in (H) were conducted between Cre(–) BM + T and Cre(+) BM + T groups. BM + T refers to bone marrow plus T cells. Data are presented as median ± interquartile range and was analyzed with the Mann-Whitney U test. NS, not significant; *$P < 0.05$; **$P < 0.01$; ***$P < 0.001$.

Figure S5. Th1 responses and endothelial injury after BMT

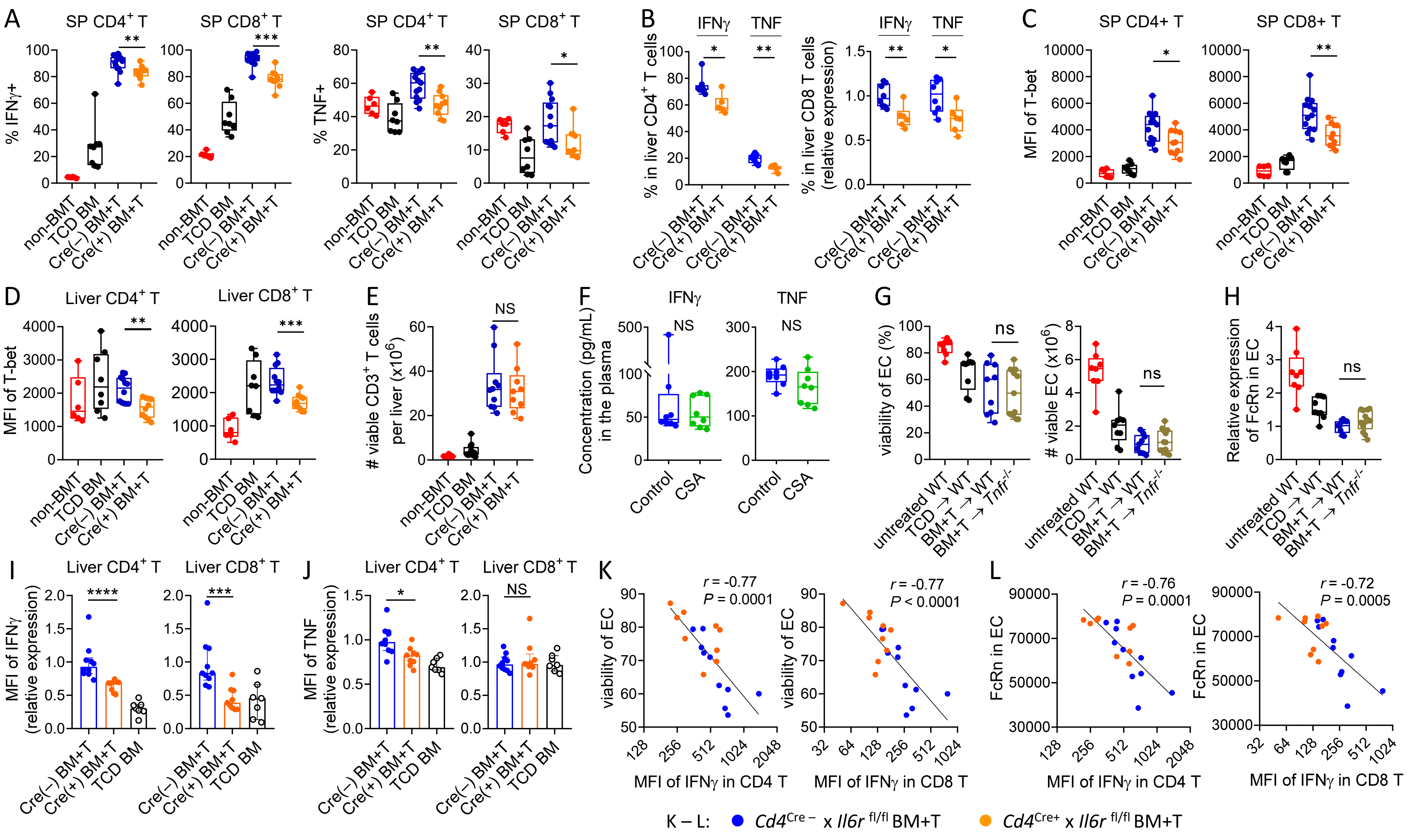

Figure S5. Th1 responses and endothelial injury after BMT

(A – E) BM ($5x10^6$) ± T cells ($2 x10^6$) from *Cd4*$^{cre+}$ x *Il6r*$^{fl/fl}$ donors (Cre+) and littermate controls (Cre–) were transplanted into B6D2F1 recipients. TCD representing BM without T cells and non-BMT controls are included for comparison. (A) Frequencies of IFNγ and TNF expression in splenic T cells on day +14 (*n* = 6, 8, 10, 13 per group from 2 experiments); (B) Expression of IFNγ and TNF in liver T cells on days 7 and 14 (*n* = 8 and 6 per group from 2 experiments); (C) Expression of T-bet (MFI) in splenic T cells on day +14 (*n* = 6, 8, 10, 13 per group from 2 experiments); (D) Expression of T-bet (MFI) in liver T cells on day +14 (n = 6, 8, 10, 12 per group from 2 experiments); (E) number of viable $CD3^+$ T cells per liver on day +14 (*n* = 8 – 12 per group from 2 experiments). (F) B6D2F1 recipients were transplanted with BM ($5x10^6$) + T cells ($2 x10^6$) from B6 donors and treated with saline (control) or CSA (5mg/kg/d) from day -1 to +13 (*n* = 8 per group from 1 experiment). Concentration of IFNγ and TNF in the plasma was determined on day +14. (G, H) Non-infected C57BL/6J (WT) or *Tnfr*$1/2^{-/-}$ recipients were transplanted with BM ($10x10^6$) ± T cells ($3x10^6$) from BALB/c donors and liver ECs were isolated on day +14 (*n* = 8, 9, 9, 13 per group from 2 experiments). (G) Viability and number of ECs and (H) relative expression of FcRn (MFI) are shown. (I – L) Transplantation was set up as described in (A – E) and livers were taken for analysis on day +21 (n = 9 – 10 for BM + T groups and n = 7 for TCD group from 2 experiments). (I, J) Expression of IFNγ and TNF in donor T cells whereby MFI of cytokine expression was normalized to Cre– BM+T groups. (K, L) Correlation between viability or FcRn expression (MFI) of EC and IFNγ expression (MFI) in donor T cells. BM + T refers to bone marrow plus T cells. Data are presented as median ± interquartile range and was analyzed with the Mann-Whitney U test. NS, not significant; *$P < 0.05$; **$P < 0.01$; ***$P < 0.001$.

Figure S6. Single cell RNA sequencing of endothelial cells

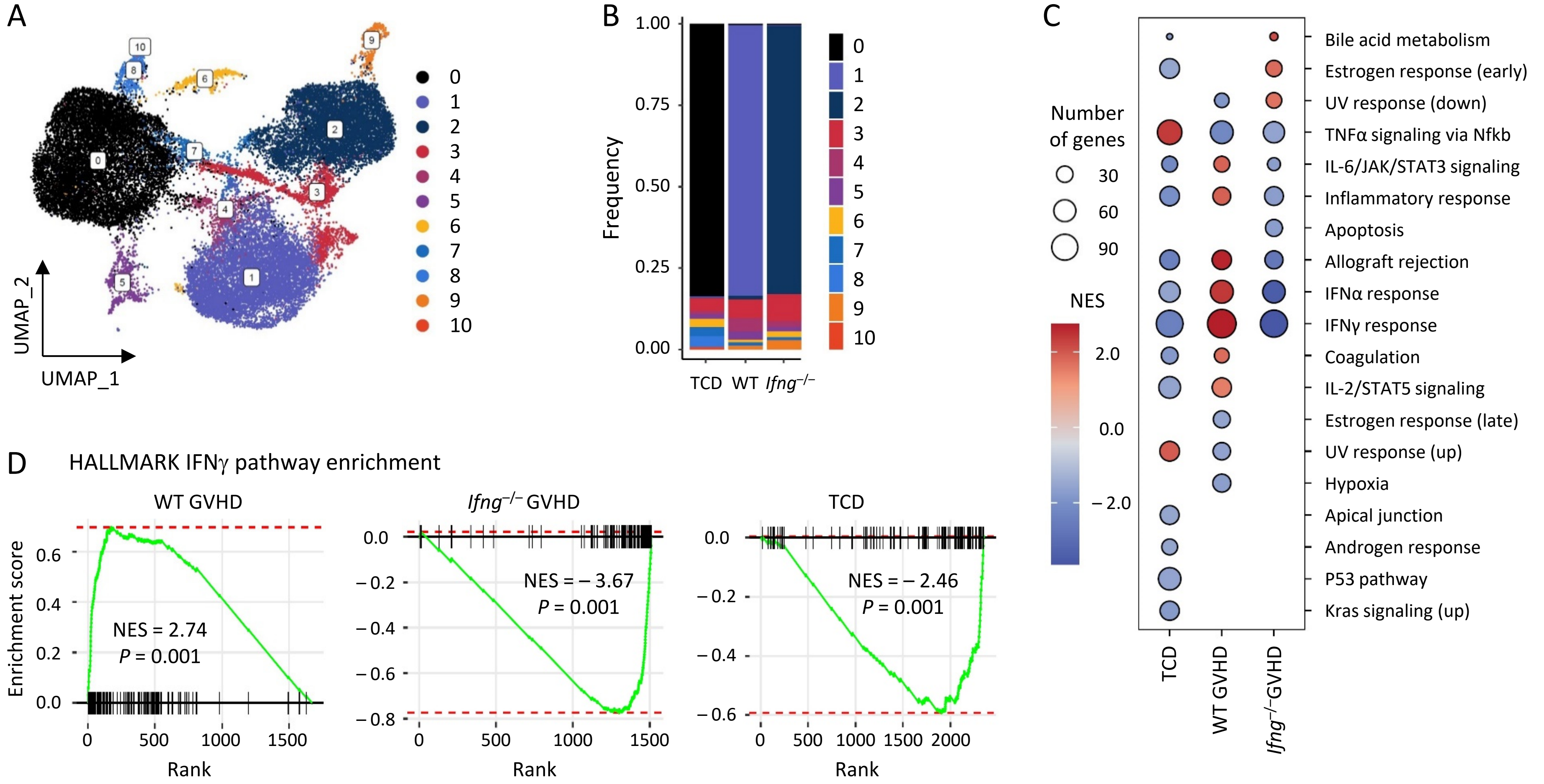

Non-infected B6D2F1 recipients were transplanted with WT TCD BM (5x10^6) ± WT or *Ifng*$^{-/-}$ T cells (2x10^6). TCD represents BM without T cells. ECs were isolated from liver on day +7, pooled from each group (TCD, WT GVHD and *Ifng*$^{-/-}$ GVHD; n = 3 per group) and processed for single cell RNAseq. (A) UMAP of ECs colored by clusters. (B) Barplot of cluster frequency across groups. (C) Hallmark pathway gene set enrichment across groups. (D) Hallmark IFNγ response pathway gene set enrichment across groups.

Figure S7. Divergent effects of IL-6 inhibition after allogeneic BMT

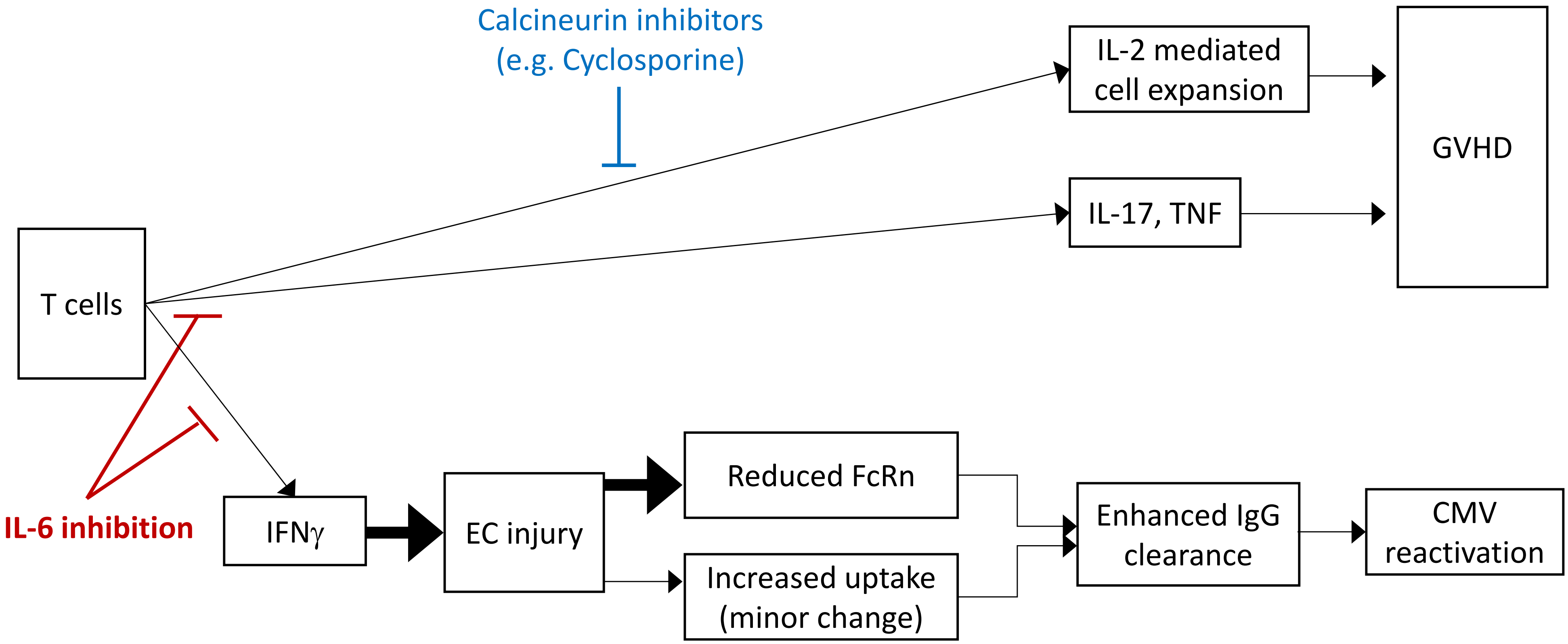

T cell derived proinflammatory cytokines (e.g. TNF and IL-17) contribute to GVHD after allogeneic BMT which can be attenuated by IL-6 inhibition. In addition, IL-6 inhibition attenuates IFNγ-dependent, T cell-mediated EC injury leading to preservation of existing recipient IgG and reduction of CMV reactivation. In contrast, calcineurin inhibitors (e.g. cyclosporine) prevent GVHD by suppressing expansion of allogeneic T cells mainly through reduced production of IL-2 but have minimal effect on EC mediated IgG loss.
